# DravidianCodeMix: sentiment analysis and offensive language identification dataset for Dravidian languages in code-mixed text

**DOI:** 10.1007/s10579-022-09583-7

**Published:** 2022-02-04

**Authors:** Bharathi Raja Chakravarthi, Ruba Priyadharshini, Vigneshwaran Muralidaran, Navya Jose, Shardul Suryawanshi, Elizabeth Sherly, John P. McCrae

**Affiliations:** 1 Insight SFI Research Centre for Data Analytics, Data Science Institute, National University of Ireland Galway, Galway, Ireland; 2 ULTRA Arts and Science College, Madurai, Tamil Nadu India; 3 School of Computer Science and Informatics, Cardiff University, Cardiff, UK; 4 Indian Institute of Information Technology and Management-Kerala, Kazhakkoottam, Kerala India

**Keywords:** Dravidian languages, Sentiment analysis, Offensive language identification, Tamil, Kannada, Malayalam, Code-mixed, Corpora

## Abstract

This paper describes the development of a multilingual, manually annotated dataset for three under-resourced Dravidian languages generated from social media comments. The dataset was annotated for sentiment analysis and offensive language identification for a total of more than 60,000 YouTube comments. The dataset consists of around 44,000 comments in Tamil-English, around 7000 comments in Kannada-English, and around 20,000 comments in Malayalam-English. The data was manually annotated by volunteer annotators and has a high inter-annotator agreement in Krippendorff’s alpha. The dataset contains all types of code-mixing phenomena since it comprises user-generated content from a multilingual country. We also present baseline experiments to establish benchmarks on the dataset using machine learning and deep learning methods. The dataset is available on Github and Zenodo.

## Introduction

Sentiment^1^^,^ 2analysis is the classification task of mining sentiments from natural language, which finds use in numerous applications such as reputation management, customer support, and moderating content in social media (Wilson et al. 2005; Agarwal et al. 2011; Thavareesan and Mahesan 2019, 2020a). Sentiment analysis has helped industry to compile a summary of human perspectives and interests derived from feedback or even just the polarity of comments (Pang and Lee 2004; Thavareesan and Mahesan 2020b). Offensive language identification is another classification task in natural language processing (NLP), where the aim is to moderate and minimise offensive content in social media. In recent years, sentiment analysis and offensive language identification have gained significant interest in the field of NLP.

Social media websites and product review forums provide opportunities for users to create content in informal settings. Moreover, to improve user experience, these platforms ensure that the user communicates his/her opinion in such a way that he/she feels comfortable either using native language or switching between one or more languages in the same conversation (Vyas et al. 2014). However, most NLP systems are trained on languages in formal settings with proper grammar, which creates issues when it comes to the analysis phase of “user generated” comments (Chanda et al. 2016; Pratapa et al. 2018). Further, most of the developments in sentiment analysis and offensive language identification systems are performed on monolingual data for high-resource languages, while the user-generated content in under-resourced settings are often mixed with English or other high-resource languages (Winata et al. 2019; Jose et al. 2020).

Code-mixing or code-switching is the alternation between two or more languages at the level of the document, paragraph, comments, sentence, phrase, word or morpheme. It is a distinctive aspect of conversation or dialogue in bilingual and multilingual societies (Barman et al. 2014). It is motivated by structural, discourse, pragmatic and socio-linguistic reasons (Sridhar 1978). Most of the social media comments are code-mixed, while the resources created for sentiment analysis and offensive language identification are primarily available for monolingual texts. Code-mixing is a common phenomenon in all kinds of communication among multilingual speakers including both speech and text-based interactions. Code-mixing refers to the way a bilingual/ multilingual speaker changes his or her utterance into another language. The vast majority of language pairs are under-resourced with regards to code-mixing tasks (Bali et al. 2014; Jose et al. 2020).

In this paper, we describe the creation of a corpus for Dravidian languages in the context of sentiment analysis and offensive language detection tasks. Dravidian languages are spoken mainly in the south of India (Chakravarthi et al. 2020c). The four major literary languages belonging to the language family are Tamil (ISO 639-3: tam), Telugu (ISO 639-3: tel), Malayalam (ISO 639-3: mal), and Kannada (ISO 639-3: kan). Tamil, Malayalam and Kannada fall under the South Dravidian subgroup while Telugu belongs to the South Central Dravidian subgroup (Vikram and Urs 2007). Each of the four languages has official status as one of the 22 scheduled languages recognised by the Government of India. Tamil also has official status in Sri Lanka and Singapore (Thamburaj and Rengganathan 2015). Although the languages are widely spoken by millions of people, the tools and resources available for building robust NLP applications are under-developed for these languages.

Dravidian languages are highly agglutinating languages and each language uses its own script (Krishnamurti 2003; Sakuntharaj and Mahesan 2016, 2017). The writing system is a phonemic abugida written from left to right for Malayalam and Kannada. The Dravidian languages scripts are first attested in the 580 BCE as Tamili^3^ script inscribed on the pottery of Keezhadi, Sivagangai and Madurai district of Tamil Nadu, India (Sivanantham and Seran 2019)^4^ by Tamil Nadu State Department of Archaeology and Archaeological Survey of India. Historically, Tamil writing system has its origin in the Tamili script that was neither purely Abugida, nor Abjad, nor Alphabet system. The writing system of Tamili was explained in the old grammar text Tolkappiyam which dates are various proposed between 9th century BCE to 6nd century BCE (Pillai 1904; Swamy 1975; Zvelebil 1991; Takahashi 1995) and in the Jaina work Samavayanga Sutta and Pannavana Sutta, these two Jain works date to 3rd-4th century BCE (Salomon 1998). At different points of time in history, Tamil was written using Tamili, Vattezhuthu, Chola, Pallava and Chola-Pallava scripts. The modern Tamil script descended from the Chola-Pallava script that became the norm in the northern part of the Tamil country around 8th century CE (Mahadevan 2003). The Malayalam script is based on the Vatteluttu script developed from old Vatteluttu with additional letters from Grantha script to write loan words (Thottingal 2019). The scripts of Kannada and Telugu had their origins from Bhattiprolu script, a southern variety of Brahmi script. From Bhattiprolu script evolved an early form of Kannada script called Kadamba script (Gai 1996) which gave rise to Telugu and Kannada scripts. Although the languages have their own scripts, social media users often use the Latin script for typing in these languages due to its ease of use and accessibility in handheld devices and computers (Thamburaj et al. 2015).

Monolingual datasets are available for Indian languages for various research aims (Agrawal et al. 2018; Thenmozhi and Aravindan 2018; Kumar et al. 2020). However, there have been few attempts to generate datasets for Tamil, Kannada and Malayalam code-mixed text (Chakravarthi et al. 2020b, c; Chakravarthi 2020; Chakravarthi and Muralidaran 2021). We believe it is essential to come up with approaches to tackle this resource bottleneck so that these languages can be equipped with NLP support in social media in a way that is both cost-effective and rapid. To create resources for a Tamil-English, Kannada-English and Malayalam-English code-mixed scenario, we collected comments on various Tamil, Kannada and Malayalam movie trailers from YouTube.

The contributions of this paper are:

1. We present the dataset for three Dravidian languages, namely Tamil, Kannada, and Malayalam, for sentiment analysis and offensive language identification tasks.
2. The dataset contains all types^5^ of code-mixing. This is the first Dravidian language dataset to contain all types of code-mixing, including mixtures of these scripts and the Latin script. The dataset consists of around 44,000 comments in Tamil-English, around 7000 comments in Kannada-English, and around 20,000 comments in Malayalam-English.
3. We provide an experimental analysis of logistic regression, naive Bayes, decision tree, random forest, SVM, BERT, DistilBERT, ALBERT, RoBERTa, XLM, XLM-R and Character BERT on our code-mixed data for classification tasks in order to create a benchmark for further research.

## Related work

Sentiment analysis helps to understand the polarity (positive, negative or neutral) of the audience towards a content (comment, tweet, image, video) or an event (Brexit, presidential elections). This data on polarity can help in understanding public opinion. Furthermore, the inclusion of sentiment analysis can improve the performance of tasks such as recommendation system (Krishna et al. 2013; Musto et al. 2017), and hate speech detection (Gitari et al. 2015). Over the last 20 years, social media networks have become a rich data source for sentiment analysis (Clarke and Grieve 2017; Tian et al. 2017). Extensive research has been done for sentiment analysis of monolingual corpora such as English (Hu and Liu 2004; Wiebe et al. 2005; Jiang et al. 2019), Russian (Rogers et al. 2018), German (Cieliebak et al. 2017), Norwegian (Mæhlum et al. 2019) and Indian languages (Agrawal et al. 2018; Rani et al. 2020). In initial research works, n-gram features were used widely for classification of sentiments (Kouloumpis et al. 2011). However recently, due to readily available data on social media, these traditional techniques have been replaced by deep neural network techniques. Patwa et al. (2020) conducted sentiment analysis on code-mixed social media text for Hindi-English and Spanish-English languages. However, sentiment analysis in Dravidian languages is under-studied.

The use of aggressive, hateful or offensive language online has proliferated in social media posts because of various technological and sociological reasons.This downturn has encouraged the development of automatic moderation systems. These systems if trained on proper data can help detect aggressive speech thus moderating spiteful content on a public platform. Collection of such data has become a crucial part of social media analysis. To facilitate the researchers working on these problems, there have been shared tasks conducted on aggression identification in social media (Kumar et al. 2018) and offensive language identification (Zampieri et al. 2019) by providing necessary datasets. As English is a commonly used language on social media, a significant amount of research goes into the identification of offensive English text. However, many internet users prefer the use of their native languages. This has given rise to the development of offensive language identification dataset in Arabic, Danish, Greek, and Turkish languages (Zampieri et al. 2020). Inspired by this we developed resources for offensive language identification for Dravidian languages.

In the past few years, cheaper internet and increased use of smartphones have significantly increased social media interaction in code-mixed native languages. Dravidian language speakers (who are often bilingual with English as it is an official language in India) with a population base of 237 million^6^ contribute to large portion of such interactions. Hence, there is an ever-increasing need for the analysis of code-mixed text in Dravidian languages. However, the number of freely available code-mixed dataset (Ranjan et al. 2016; Jose et al. 2020) are still limited in number, size, and availability. Sowmya Lakshmi and Shambhavi (2017) developed a Kannada-English dataset containing English and Kannada text with word-level code-mixing. Also, they employed a stance detection system to detect stance in Kannada-English code-mixed text (on social media) using sentence embeddings. Shalini et al. (2018) have used distributed representations for sentiment analysis of Kannada-English code-mixed texts through neural networks, which had three tags: Positive, Negative and Neutral. However, the dataset for Kannada was not readily available for research purposes. To give motivation for further research we conducted (Chakravarthi et al. 2020a, d; Mandl et al. 2020; Chakravarthi et al. 2021) a shared task that provided Tamil-English, Kannada-English, and Malayalam-English code-mixed datasets using which participants trained models that identify the sentiments (task A) and offensive classes (task B) in both the languages.

Most of the recent studies on sentiment analysis and offensive language identification have been conducted on high-resourced languages from social media platforms. Models trained on such highly resourced monolingual data have succeeded in predicting sentiment and offensiveness. However, with the increased social media usage of bilingual users, a system trained on under-resourced code-mixed data is needed. In spite of this need, no large datasets for Tamil-English, Kannada-English and Malayalam-English are available. Hence, inspired by Severyn et al. (2014), we collected and created a code-mixed dataset from YouTube. In this work, we describe the process of corpora creation for under-resourced Dravidian languages from YouTube comments. This is an extension of two workshop papers (Chakravarthi et al. 2020b, c) and shared tasks (Chakravarthi et al. 2020d). We present DravidianCodeMix corpora for Tamil-English (40,000 + comments), Kannada-English (7000 + comments) and Malayalam-English (nearly 20,000 comments) with manually annotated labels for sentiment analysis and offensive language identification. We used Krippendorff’s alpha to calculate agreement amongst annotators. We made sure that each comment is annotated by at least three annotators and made the labelled corpora freely available for research purpose. For bench marking, we provided baseline experiments and results on ’DravidianCodeMix’ corpora using machine learning models.

**Fig. 1:**
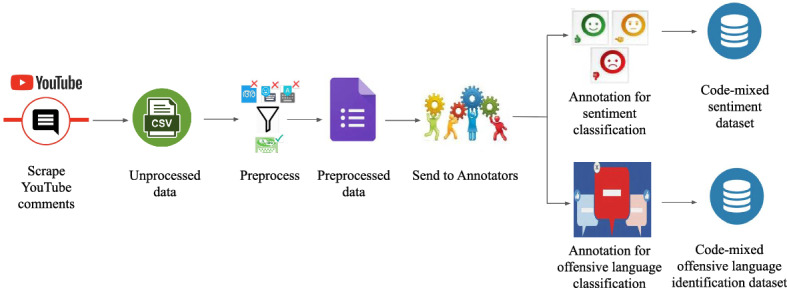
Data collection process

**Fig. 2:**
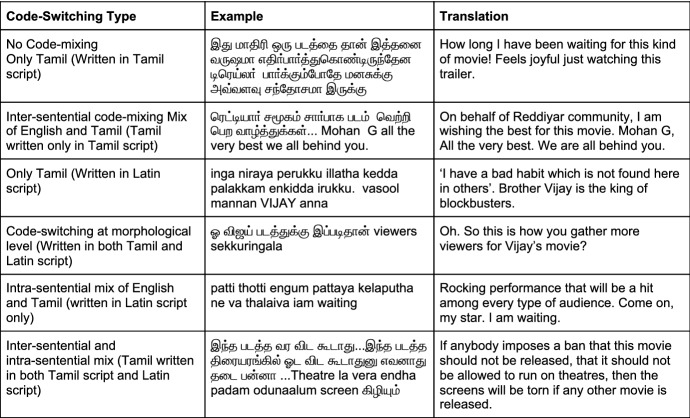
Examples of code mixing in Tamil dataset

**Fig. 3:**
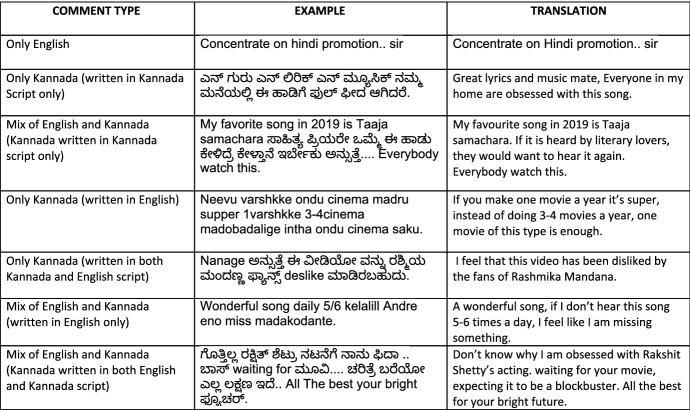
Examples of code mixing in Kannada dataset

**Fig. 4:**
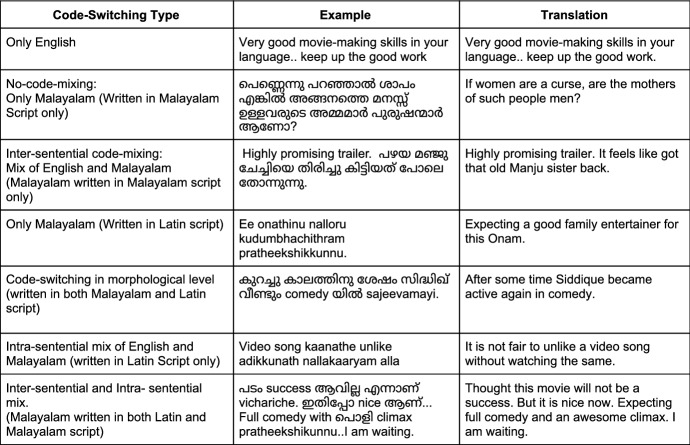
Examples of code mixing in Malayalam dataset

## Raw data

Online media, for example, Twitter, Facebook or YouTube, contain quickly changing data produced by millions of users that can drastically alter the reputation of an individual or an association. This raises the significance of programmed extraction of sentiments and offensive language used in online social media. YouTube is one of the popular social media platforms in the Indian subcontinent because of the wide range of content available from the platform such as songs, tutorials, product reviews, trailers and so on. YouTube allows users to create content and other users to comment on the content. It allows for more user-generated content in under-resourced languages. Hence, we chose YouTube to extract comments to create our dataset. We chose movie trailers as the topic to collect data because movies are quite popular among the Tamil, Malayalam, and Kannada speaking populace. This increases the chance of getting varied views on one topic. Figure 1 shows the overview of the steps involved in creating our dataset.

We compiled the comments from different film trailers of Tamil, Kannada, and Malayalam languages from YouTube in the year 2019. The comments were gathered using *YouTube Comment Scraper tool*^7^. We utilized these comments to make the datasets for sentiment analysis and offensive language identification with manual annotations. We intended to collect comments that contain code-mixing at various levels of the text, with enough representation for each sentiment and offensive language classes in all three languages. It was a challenging task to extract the necessary text that suited our intent from the comment section, which was further complicated by the presence of remarks in other non-target languages. As a part of the preprocessing steps to clean the data, we utilized *langdetect library*^8^ to tell different languages apart and eliminate the unintended languages. The Langdetect library, however, is a script detection library that filters out languages based on certain scripts. This has serious limitations as it misses out a number of languages written in non-conventional script. This explains why we still get data from other languages despite using this library. Examples of code-mixing in Tamil, Kannada and Malayalam corpora are shown in Figs. 2, 3, and 4 along with their translations in English. By keeping data privacy in mind, we made sure that all the user-related information is removed from the corpora. As a part of the text-preprocessing, we removed redundant information such as URL.

Since we collected corpora from social media, our corpora contain different types of real-world code-mixed data. Inter-sentential switching is characterised by change of language between sentences where each sentence is written or spoken in one language. Intra-sentential switching occurs within a single sentence, say one of the clause is in one language and the other clause is in the second language. Our corpora contains all forms of code-mixing ranging from purely monolingual texts in native languages to mixing of scripts, words, morphology, inter-sentential and intra-sentential switches. We retained all the instances of code-mixing to faithfully preserve the real-world usage.

**Fig. 5:**
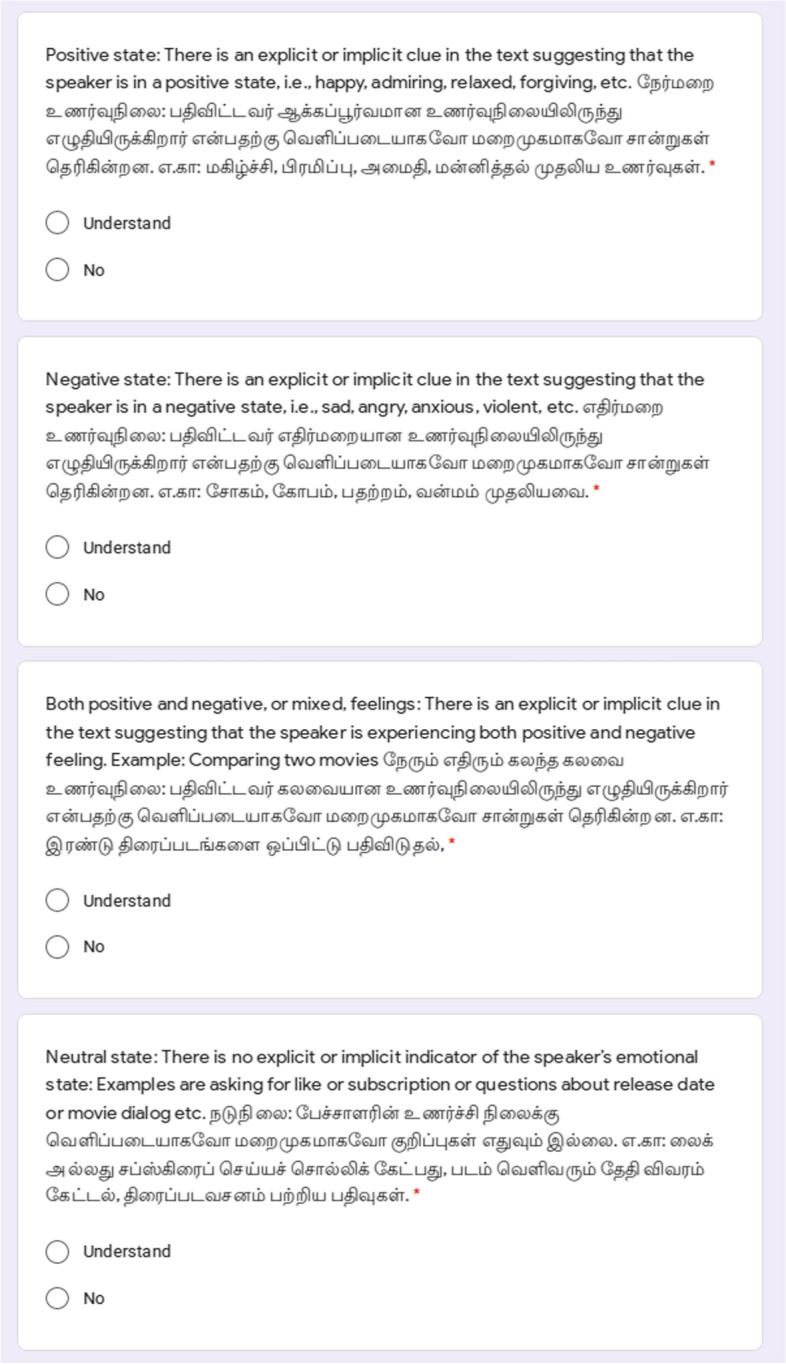
Example Google Form with annotation instructions for sentiment analysis

## Methodology of annotation

We create our corpora for two tasks, namely, sentiment analysis and offensive language identification. We anonymized the data gathered from Youtube in order to protect user privacy.

### Annotation process

In order to find volunteers for the annotation process, we contacted students in Indian Institute of Information Technology and Management-Kerala for Malayalam, Indian Institute of Information Technology-Tiruchirapalli and Madurai Kamaraj University for Tamil. For Kannada, we contacted students in Visvesvaraya College of Engineering, Bangalore University. The student volunteer annotators received the link to a Google Form and did the annotations on their personal computers. The authors’ family members also volunteered to annotate the data. We created Google Forms to gather annotations from annotators. Information on gender, education background and medium of schooling were collected to know the diversity of the annotators. The annotators were cautioned that the user remarks may have hostile language. They were given a provision to discontinue with the annotation process in case the content is too upsetting to deal with. They were asked not to be partial to a specific individual, circumstance or occasion during the annotation process. Each Google form had been set to contain up to 100 comments and each page was limited to contain ten comments. The annotators were instructed to agree that they understood the scheme before they were allowed to proceed further. The annotation setup involved three stages. To begin with, each sentence was annotated by two individuals. In the second step, the data was included in the collection if both the annotations agreed. In the event of contention, a third individual was asked to annotate the sentence. In the third step, in the uncommon case that all the three of them disagreed, at that point, two additional annotators were brought in to label the sentences. Each form was annotated by at least three annotators.

### Sentiment analysis

For sentiment analysis, we followed the methodology taken by Chakravarthi et al. (2020c), and involved at least three annotators to label each sentence. The following annotation schema was given to the annotators in English and Dravidian languages.

- **Positive state:** Comment contains an explicit or implicit clue in the content recommending that the speaker is in a positive state.
- **Negative state:** Comment contains an explicit or implicit clue in the content recommending that the speaker is in a negative state.
- **Mixed feelings:** Comment contains an explicit or implicit clue in both positive and negative feeling.
- **Neutral state:** Comment does not contain an explicit or implicit indicator of the speaker’s emotional state.
- **Not in intended language:** If the comment is not in the intended language. For example, for Tamil, if the sentence does not contain Tamil written in Tamil script or Latin script, then it is not Tamil. These comments were discarded after the data annotation process.

**Fig. 6:**
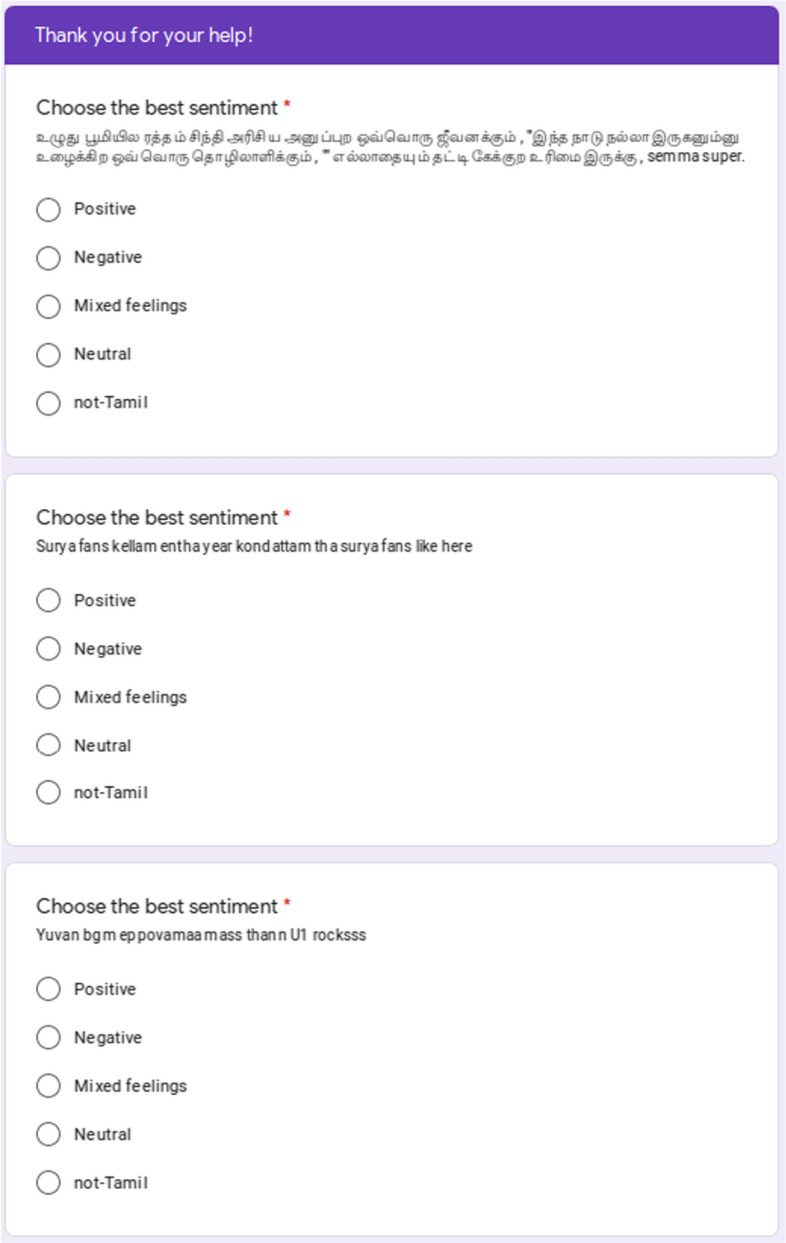
Examples from the first page of the Google form for sentiment analysis

Figures 5 and 6 show the sample Google Forms for general instructions and sentiment analysis respectively.

### Offensive language identification

We constructed an offensive language identification dataset for Dravidian languages by adapting the work of Zampieri et al. (2019). We reduced the three-level hierarchical annotation scheme of this work into a flat scheme with five labels to account for the types of offensiveness in the comments and the sixth label **Not in intended language** accounts for comments written in a language other than the intended language. Examples for this are the comments written in other Dravidian languages using Roman script. To simplify the annotation decisions, the six categories into which each comment will be split into are as follows:

- **Not Offensive**: Comment does not contain offence or profanity.
- **Offensive Untargeted**: Comment contains offence or profanity not directed towards any target. These are the comments which contain unacceptable language without targeting anyone.
- **Offensive Targeted Individual**: Comment contains offence or profanity which targets an individual.
- **Offensive Targeted Group**: Comment contains offence or profanity which targets a group or a community.
- **Offensive Targeted Other**: Comment contains offence or profanity which does not belong to any of the previous two categories (e.g. a situation, an issue, an organization or an event).
- **Not in indented language**: If the comment is not in the intended language. For example, in Tamil task, if the sentence does not contain Tamil written in Tamil script or Latin script, then it is not Tamil. These comments were discarded after the data annotation process.

**Fig. 7:**
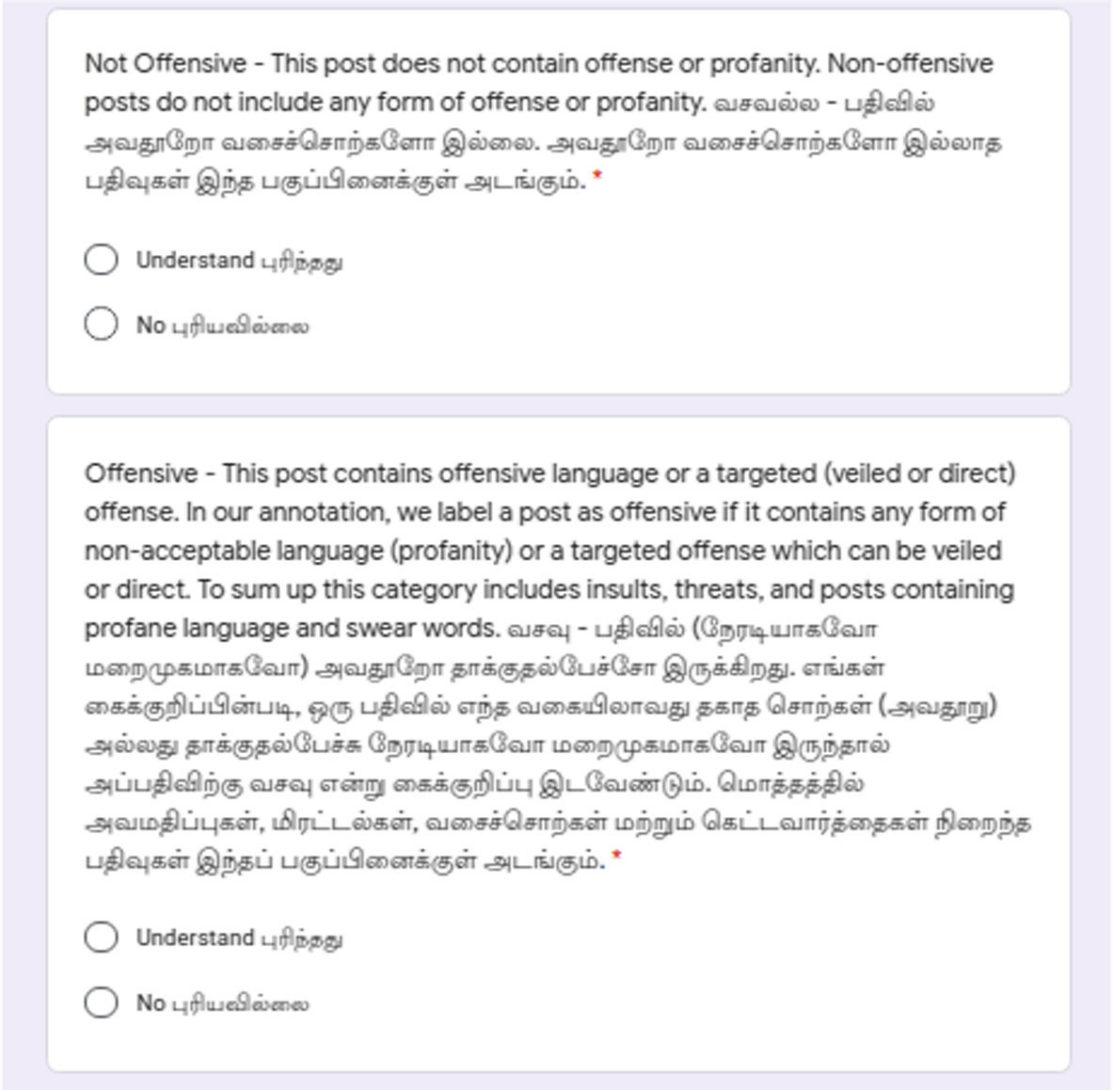
Example Google Form with annotation instructions for offensive language identification

**Fig. 8:**
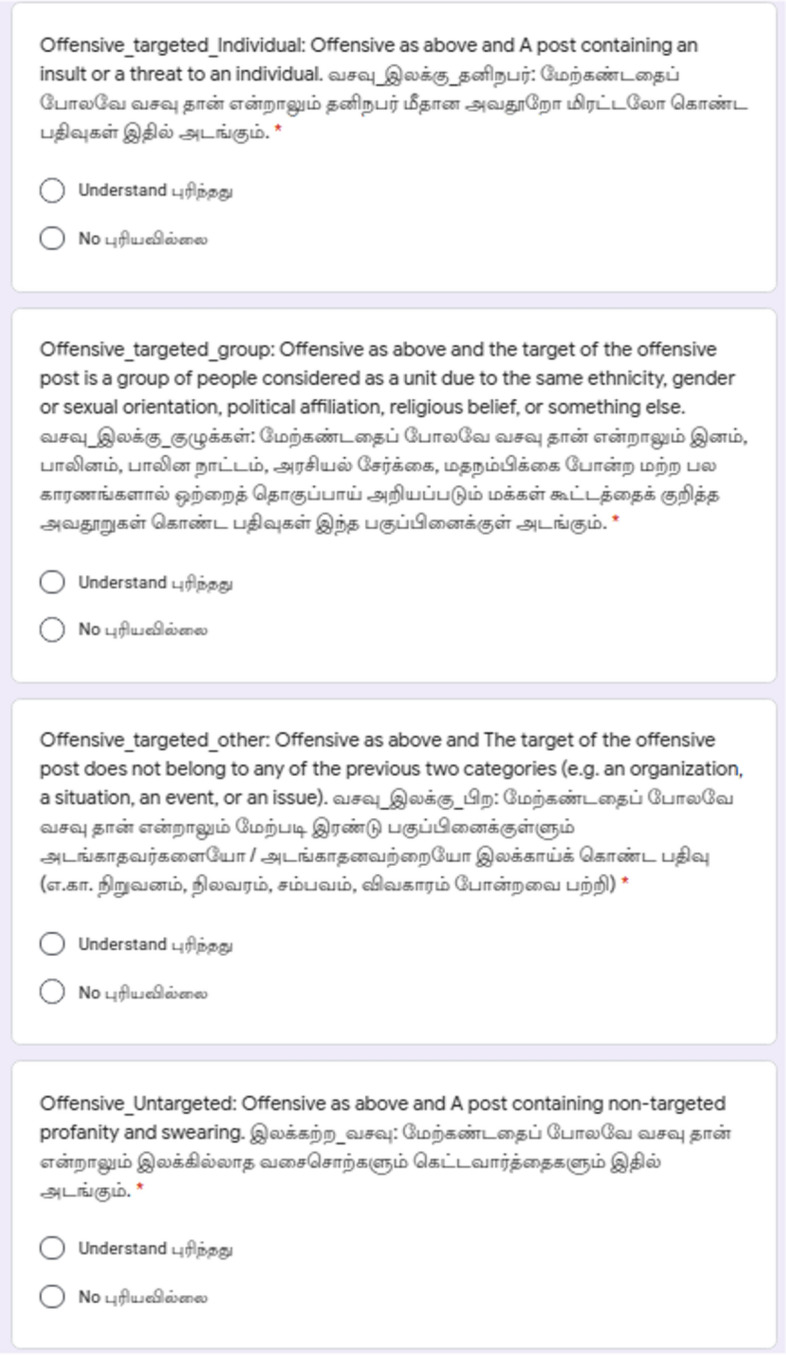
Example Google Form with annotation instructions for offensive language identification

**Fig. 9:**
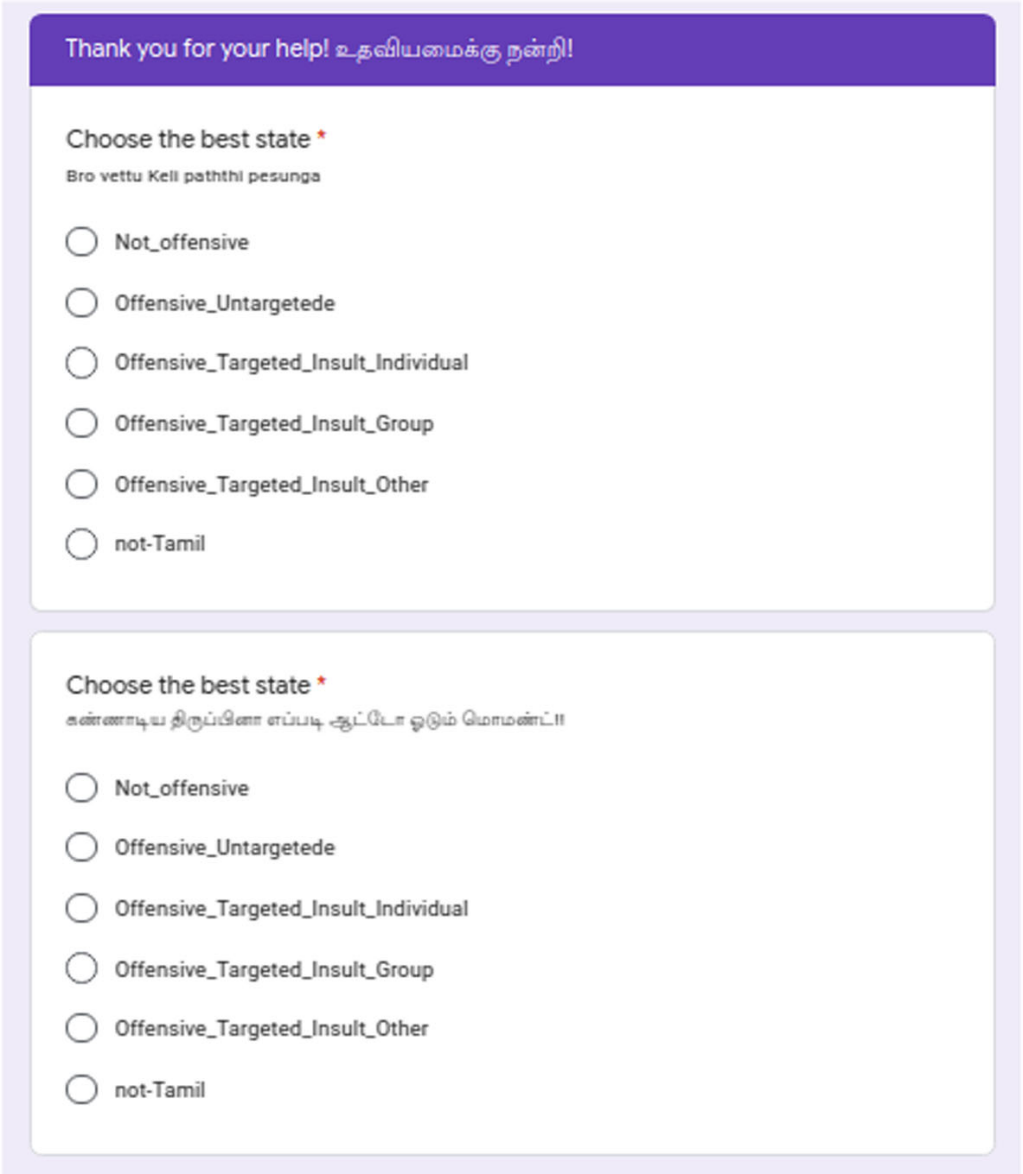
Examples from the first page of the Google Form for offensive language identification

Examples of the Google Forms in English and native language for offensive language identification task are given in Figs. 7, 8, and 9.

**Table 1:** Annotators statistics for sentiment analysis

Language		Tamil	Malayalam	Kannada
Gender	Male	9	2	2
	Female	2	4	3
	Non-binary	0	0	0
Higher education	Undegraduate	2	0	1
	Graduate	2	0	2
	Postgraduate	7	6	2
Medium of schooling	English	6	5	4
	Native language	5	1	1
Total		11	6	5

**Table 2:** Annotators statistics for offensive language identification

Language		Tamil	Malayalam	Kannada
Gender	Male	6	2	3
	Female	6	4	2
	Non-binary	0	0	0
Higher education	Undegraduate	2	0	0
	Graduate	5	0	3
	Postgraduate	5	6	2
Medium of schooling	English	6	5	3
	Native language	7	1	2
Total		12	6	5

Once the Google Form was ready, we sent it out to an equal number of males and females to enquire their willingness to annotate. We got varied responses from them and so our distribution of male and female annotators involved in the task are different. From Table 1, we can see that only two female annotators volunteered to contribute for Tamil while there were more female annotators for Malayalam and Kannada. For offensive language identification, we can see that there is a balance in gender from Table 2. The majority of the annotators have received postgraduate level of education. We were not able to find volunteers of non-binary gender to annotate our dataset. All the annotators who volunteered to annotate the Tamil-English, Kannada-English and Malayalam-English datasets had bilingual proficiency in the respective code-mixed pairs and they were prepared to take up the task seriously. From Table 1 and 2, we can observe that the majority of the annotators’ medium of schooling is English even though their mother tongue is Tamil, Kannada or Malayalam. For Kannada and Malayalam languages only one annotator from each language received their education through the medium of their native language. Although the medium of education of the participants was skewed towards the English language, we were careful it would not affect the annotation task by ensuring that all of them are fully proficient in using their native language.

We were aware that there could be other factors affecting the annotation decisions on offensive language such as the annotators’ age, their field of education and their ideological stance. Due to privacy issues involved, we did not collect this information from annotators. A sample form (first assignment) was annotated by experts and a gold standard was created. The experts were a team of NLP researchers who have experience working with creating annotation standards and guidelines. We manually compared the gold standard annotations with the volunteer submission form. To control the quality of annotation, we eliminated the annotators whose label assignments in the first form were not good. For instance, if the annotators showed an unreasonable delay in responding or if they labelled all sentences with the same label or if more than fifty annotations in a form were wrong, we eliminated those contributions. A total of 22 volunteers and 23 volunteers, for sentiment analysis and offensive language identification tasks respectively, were involved in the process. Once they filled up the Google Form, 100 sentences were sent to them. If an annotator offered to volunteer more, the next Google Form was sent to them with another set of 100 sentences and in this way each volunteer chose to annotate as many sentences from the corpus as they wanted. We sent out the same comment forms to annotators but some of the forms were incomplete so we discarded them. Hence there is some difference between the sentiment dataset and offensive dataset. However, there is more than 98% comments overlap between sentiment dataset and offensive dataset.

**Table 3:** Inter-annotator agreement in Krippendorff’s alpha

	Sentiment analysis		Offensive language identification	
	Nominal	Ordinal	Nominal	Ordinal
Tamil	0.6735	0.6534	0.7452	0.7634
Malayalam	0.8753	0.8463	0.8345	0.8374
Kannada	0.7356	0.7465	0.8456	0.8443

### Inter-annotator agreement

Inter-annotator agreement is a measure of the extent to which the annotators agree in their rating. This is necessary to ensure that the annotation scheme is consistent and that different raters are able to assign the same sentiment label to a given comment. There are two questions related to inter-annotator agreement: How do the annotators agree or disagree in their annotation? How much of the observed agreement or disagreement among the annotators might be due to chance? While the percentage of agreement is fairly straightforward, answering the second question involves defining and modelling what chance is and how to measure the agreement due to chance. There are different inter-annotator agreement measures that are intended to answer this in order to measure the reliability of the annotation. We utilized **Krippendorff’s alpha** $(\alpha )$ (Krippendorff 1970) to gauge the agreement between annotators because of the nature of our annotation setup. Krippendorff’s alpha is a rigorous statistical measure that accounts for incomplete data and, consequently, does not require every annotator to annotate every sentence. It is also a measure that considers the level of disagreement between the anticipated classes, which is critical in our annotation scheme. For example, if the annotators differ among **Positive** and **Negative** class, this difference is more genuine than when they differ between **Mixed feelings** and **Neutral state**. $\alpha$ is sensitive to such disagreements. $\alpha$ is characterized by:

1 $$\begin{aligned} \alpha = 1 - \frac{D_o}{D_e} \end{aligned}$$

$D_o$ is the observed disagreement between sentiment labels assigned by the annotators and $D_e$ is the disagreement expected when the coding of sentiments can be attributed to chance rather than due to the inherent property of the sentiment itself.

2 $$\begin{aligned} D_o= & {} \frac{1}{n}\sum _{c}\sum _{k}o_{ck\;metric}\;\delta ^2_{ck} \end{aligned}$$

3 $$\begin{aligned} D_e= & {} \frac{1}{n(n-1)} \sum _{c}\sum _{k}n_c \; .\;n_{k\;metric}\,\delta ^2_{ck} \end{aligned}$$

Here $o_{ck}\;n_c\;n_k\;$ and *n* refer to the frequencies of values in the coincidence matrices and *metric* refers to any metric or level of measurement such as nominal, ordinal, interval, ratio and others. Krippendorff’s alpha applies to all these metrics. We used nominal and ordinal metric to calculate inter-annotator agreement. The range of $\alpha$ is between ‘0’ and ‘1’, $1 \ge \alpha \ge 0$. When $\alpha$ is ‘1’ there is perfect agreement between the annotators and when ‘0’ the agreement is entirely due to chance. Care should be taken in interpreting the reliability of the results shown by Krippendorf’s alpha because reliability basically measures the amount of noise in the data. However, the location of noise and the strength of the relationship measured will interfere with the reliability of the estimate. It is customary to require $\alpha$ $\ge$ .800. A reasonable rule of thumb that allows for tentative conclusions to be drawn requires $0.67 \le \alpha \le 0.8$ while $\alpha \ge$ .653 is the lowest conceivable limit. We used *nltk*^9^ for calculating Krippendorff’s alpha $(\alpha )$. The results of inter-annotator agreement between our annotators for different languages on both sentiment analysis and offensive language identification tasks are shown in Table 3.

## Corpus statistics

Tables 4 and 5 show the text statistics (number of words, vocabulary size, number of comments, number of sentences, and average number of words per sentences) for sentiment analysis and offensive language identification for Tamil, Malayalam and Kannada. The Tamil dataset had the highest number of samples while Kannada had the least on both the tasks. On average, each comment contained only one sentence.

**Fig. 10:**
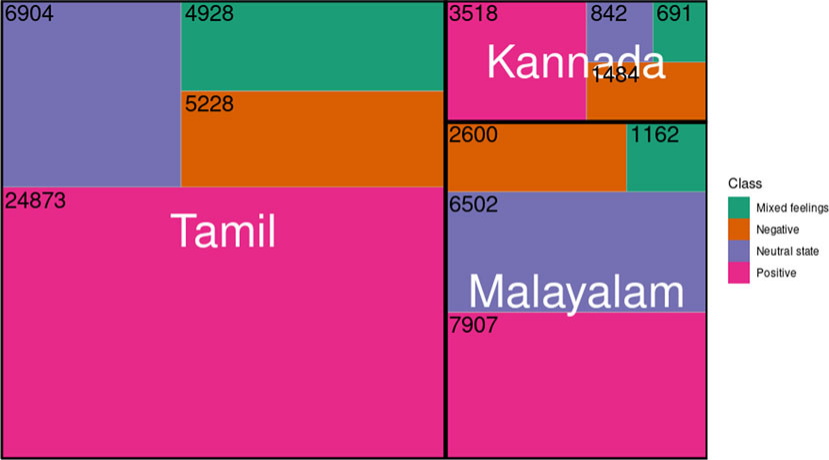
Treemap for comparing sentiment classes across Tamil, Malayalam and Kannada

**Fig. 11:**
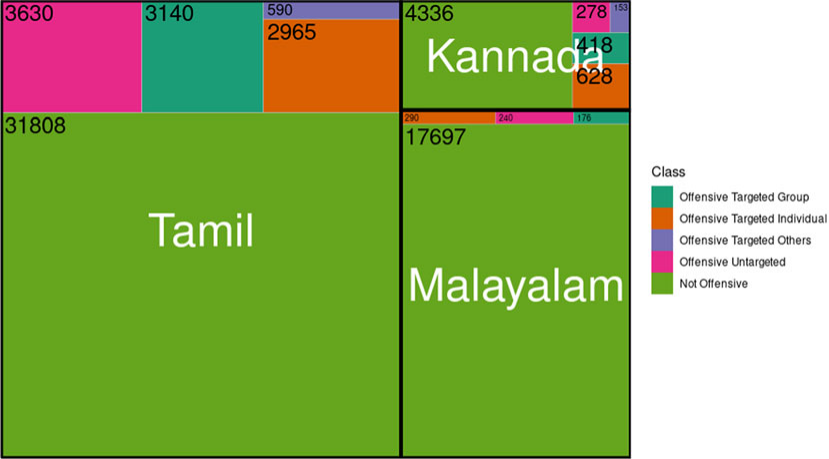
Treemap for comparing offensive classes across Tamil, Malayalam and Kannada

**Fig. 12:**
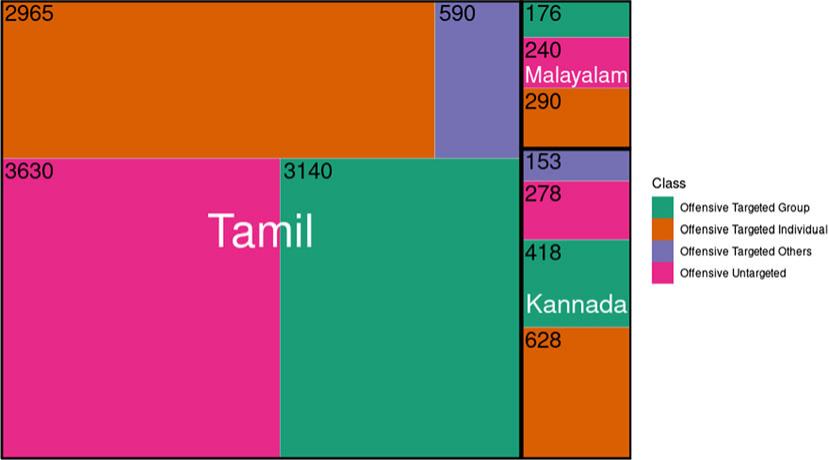
Treemap for comparing offensive classes (excluding Not Offensive class) across Tamil, Malayalam and Kannada

Table 6 and Table 7 show the class distribution across Tamil, Malayalam and Kannada in sentiment analysis and offensive language identification tasks. Furthermore, tree-maps in Figs. 10 and 11 depict the comparative analysis of distribution of sentiment and offensive classes across languages. Figure 10 illustrates that there are more number of samples labelled “Positive” than any other class in all the languages. While the disparity between “Positive” and other classes is large in Tamil, it is not the case with Malayalam and Kannada. In Malayalam, “Neutral state” is the second-largest class in terms of distribution; 6502 number of comments labelled “Neutral state” could mean that most of the comments in Malayalam are vague remarks as the sentiment behind them is unknown. On the other hand, Kannada has the least number of “Neutral state” class. Figure 11 shows that all languages have not-offensive class in the majority. In the case of Tamil, 75.49% of the total comments are not offensive, while Malayalam has 96.16% non-offensive comments. But there is no consistent trend observable amongst offensive classes across the languages shown in Fig. 12. In the case of Tamil, 60% of the offensive comments are targeted (group or individual). Similar trends are seen in the case of Malayalam (66%) and Kannada (81.17%). Absence (Malayalam) or least (Tamil, Kannada) number of targeted other category comments points to the fact that most of the offensive comments are targeted towards either an individual or a group.

**Table 4:** Corpus statistics for sentiment analysis

Language	Tamil	Malayalam	Kannada
Number of words	456,586	202,305	56,665
Vocabulary size	105,043	61,215	22,200
Number of comments	41,933	18,171	6535
Number of sentences	64,773	30,872	9751
Average number of words per sentence	11	11	8
Average number of sentences per comment	1	1	1

**Table 5:** Corpus statistics for offensive language identification

Language	Tamil	Malayalam	Kannada
Number of words	457,748	180,479	54,082
Vocabulary size	104,602	42,576	21,403
Number of comments	42,133	18,403	5874
Number of sentences	64,991	29,601	8983
Average number of words per sentence	11	10	8
Average number of sentences per comment	1	1	1

**Table 6:** Sentiment analysis dataset distribution

Class	Tamil	Malayalam	Kannada
Negative	5228 (12.46%)	2600 (14.30%)	1,484 (22.70%)
Neutral state	6904 (16.46%)	6502 (35.78%)	842 (12.88%)
Mixed feelings	4928 (11.75%)	1162 (6.39%)	691 (10.57%)
Positive	24,873 (59.31%)	7907 (43.51%)	3,518 (53.83%)
Total	41,933	18,171	6535

**Table 7:** Offensive language identification dataset distribution

Class	Tamil	Malayalam	Kannada
Not offensive	31,808 (75.49%)	17,697 (96.16%)	4336 (74.85%)
O-Untargeted	3630 (8.61%)	240 (1.30%)	278 (4.73%)
O-Targeted individual	2965 (7.03%)	290 (1.57%)	628 (10.69%)
O-Targeted group	3140 (7.45%)	176 (0.95%)	418 (7.11%)
O-Targeted others	590 (1.40%)	–	153 (2.60%)
Total	42,133	18,403	5874

*O* offensive. *O-Untargeted* offensive untargeted

Our datasets are stored in tab separated files. The first column of the tsv file contains the comments from YouTube and the second column has the final annotation.

## Difficult examples

The social media comments that form our dataset are code-mixed showing a mixture of Dravidian languages and English. This poses a few major difficulties while annotating the sentiments and offensive language categories on our dataset. Dravidian languages are under-resourced languages and the mixing of scripts makes the annotation task difficult since the annotators must have learned both the scripts, be familiar with how English words are modified to native phonology and how the meaning of certain English words have a different meaning in the given local language. Reading and understanding the code mixed text often with non-standardised spelling is difficult unless the annotator is well-versed in both the languages (Sridhar and Sridhar 1980). This created difficulty in finding volunteer annotators who were fluent in both the languages. Moreover, we have created the annotation labels with the help of volunteer annotators for three languages (not just one language). It is challenging and time consuming to collect this much amount of data from bilingual, volunteer annotators from three different language groups.

While annotating, it was found that some of the comments were ambiguous in conveying the right sentiment of the viewers. Hence the task of annotation for sentiment analysis and offensive language identification seemed difficult. The problems include the comparison of the movie with movies of same or other industries, expression of opinion of different aspects of the movie in the same sentence. Below are a few examples of such comments and details of how we resolved those issues are provided. In this section, we talk about some examples from Tamil language that were difficult to annotate.

- **Enakku iru mugan trailer gnabagam than varuthu** - *All it reminds me of is the trailer of the movie Irumugan*. Not sure whether the speaker enjoyed Irumugan trailer or disliked it or simply observed the similarities between the two trailers. The annotators found it difficult to identify the sentiment behind the comment consistently.
- **Rajini ah vida akshay mass ah irukane** - *Akshay looks more amazing than Rajini*. Difficult to decide if it is a disappointment that the villain looks better than the hero or a positive appreciation for the villain actor. Some annotators interpreted negative sentiment while some others took it as positive.
- ** Ada dei nama sambatha da dei ** - *I wonder, Is this our sampath? Hey!.* Conflict between neutral and positive.
- **Lokesh kanagaraj movie naalae.... English Rap....Song vandurum** - *If it is a movie of Lokesh kanagaraj, it always has an English rap song*. Ambiguous sentiment.
- **Ayayo bigil aprm release panratha idea iruka lokesh gaaru** - *Oh Dear! Are you even considering releasing the movie Bigil, Mr.Lokesh?*. This comment has a sinlge word ‘garu’^10^ which is a non-Tamil , non-English word borrowed from Telugu language which is a politeness marker. However, in this context the speaker uses the word sarcastically to insult the director because of the undue delay in releasing the movie. The annotators were inconsistent in interpreting this as offensive or not-Tamil.
- **No of dislikes la theriyudhu, idha yaru dislike panni irrupanga nu** - *It is obvious from the number of dislikes as to who would have disliked this (trailer).* The comment below the trailer of a movie which talks about the caste issues in contemporary Tamil society. Based on the content of the trailer, the speaker offensively implies that the scheduled caste people are the ones who would have disliked the movie and not other people. Recognising the offensive undercurrent in a seemingly normal comment is difficult and hence these examples complicate the annotation process.

According to the instructions, questions about music director, movie release date and comments containing speaker’s remarks about the date and time of watching the video should be treated as belonging to neutral class. However the above examples show that some comments about the actors and movies can be ambiguously interpreted as neutral or positive or negative. We found annotator disagreements in such sentences. Below, we give similar examples from Malayalam.

- ***Realistic bhoothanghalil ninnu oru vimochanam pratheekshikkunnu*** -*Hoping for a deliverance from realistic demons.* No category of audience can be pleased simultaneously. The widespread opinion is that the Malayalam film industry is advancing with more realistic movies. Therefore a group of audience who is more fond of action or non-realistic movies are not satisfied with this culture of realistic movies. In this comment, the viewer is not insulting this growing culture, but expecting that the upcoming film is of his favourite genre. Hence we labelled it non-offensive.
- ***Ithilum valiya jhimikki kammal vannatha*** - *There was an even bigger ‘pendant earring’.* *‘Jhimikki kammal’ * was a trending song from a movie of the same actor mentioned here. The movie received huge publicity even before its release because of the song but it turned out to be a disappointment after its release. Thus the annotators got confused whether the comment is meant as an insult or not. But we concluded that the viewer is not offending the present trailer but marks his opinion as a warning for the audience to not judge the book by its cover.
- *** Ithu kandittu nalla tholinja comedyaayi thonniyathu enikku mathram aano?***- *Am I the only person here who felt this a stupid comedy?* The meaning of the Malayalam word mentioned here corresponding to the word ‘stupid’ varies with regions of Kerala. Hence the disparity in opinion between annotators who speaks different dialects of Malayalam was evident. Though in few regions it is offensive, generally it is considered as a byword for ‘bad’.
- *** aa cinemayude peru kollam. Ithu Dileep ne udheshichanu,ayale mathram udheshichanu*** -*The name of that movie is good. It is named after Dileep and intended only for him.* It is quite obvious that there is a chance of imagining several different movie names based on the subjective predisposition of the annotator. As long as the movie name is unknown here, apparently no insult can be proved and there is no profane language used in the sentence either.
- ***Kanditt Amala Paul Aadai Tamil mattoru version aanu ennu thonnunu*** - *It looks like another version of Amala Paul’s Tamil movie Aadai.* Here the viewer doubts the Malayalam movie ‘Helen’ is similar to the Tamil movie ‘Aadai’. Though the movie ‘Aadai’ was positively received by viewers and critics, we cannot generalize and assume that this comment also as positive only because of this comparison. Hence we add it to the category of ‘mixed feeling’.
- ***Evideo oru Hollywood story varunnilleee. Oru DBT.*** -*Somewhere there is a Hollywood storyline...one doubt.* This is also a comparison comment of that same movie ‘Helen’ mentioned above. Nevertheless, here the difference is that the movie is compared with the Hollywood standard, which is well-known worldwide and is generally considered positive. Hence it is marked as a positive comment.
- ***Trailer pole nalla story undayal mathiyarinu.***-*It was good enough to have a good story like the trailer.* Here viewer mentioned about two aspects of the movie viz: ‘trailer’ and ‘story’. He appreciates the trailer but doubts the quality of the story at the same time. We considered this comment positive because it is clear that he enjoyed the trailer and conveys strong optimism for the movie.

## Benchmark systems

In this section, we report the results obtained in three languages for both the tasks in the corpora introduced above. Like many earlier studies, we approach the tasks as text classification tasks. In order to provide a simple baseline, we applied several traditional machine learning algorithms such as Logistic Regression (LR), Support Vector Machine (SVM), Multinomial Naive Bayes (MNB), K-Nearest Neigbours (KNN), Decision Trees (DT) and Random Forests (RF) separately, for both sentiment analysis and offensive language detection on the code-mixed datasets. We also conducted experiments with BERT, Character BERT, DistilBERT, RoBERTA, XLM, XLM-R on our code-mixed data for classification tasks to establish good, strong baselines (Tables 8 and 9).

**Table 8:** Train-development-test data distribution with 90–5–5% train-dev-test split for sentiment analysis

	Tamil	Malayalam	Kannada
Training	37,844	16,398	5896
Development	1992	864	310
Test	2097	909	329
Total	41,933	18,171	6535

**Table 9:** Train-development-test data distribution with 90–5–5% train-dev-test for offensive language identification

	Tamil	Malayalam	Kannada
Training	38,024	16,607	5308
Development	2002	875	276
Test	2107	921	290
Total	42,133	18,403	5874

### Experiments setup

We used 90–5–5% randomly sampled data split for training, development and test set for all the experimental setup. All the duplicated entries were removed from the dataset before the split to make test and development data truly unseen. All the experiments are tuned to the development set and tested on the test set.

#### Logistic regression (LR):

LR is one of the base-line machine learning algorithms, which is also a probabilistic classifier used for the task of classification of data (Genkin et al. 2007). This is basically the transformed version of linear regression using the logistic function (Park 2013). Accordingly it takes the real-valued features as input which is later multiplied by a weight and the sum is fed to the sigmoid function $\sigma (z)$ also called the logistic function to obtain the class probability (Shah et al. 2020). The decision is made based on the value set as threshold. Sigmoid function is as given below:

4 $$\begin{aligned} \sigma (z) = \frac{\mathrm {1} }{\mathrm {1} + e^{-z} } \end{aligned}$$

Logistic regression has a close relationship with neural networks as the latter can also be viewed as a stack of several LR classifiers (de Gispert et al. 2015). Unlike Naïve Bayes which is a generative classifier, LR is a discriminative classifier (Ng and Jordan 2002). While Naïve Bayes holds strict conditional independence assumptions, LR is evidently more robust to correlated features (Jin and Pedersen 2018). It means that when there are more than one features say F1,F2,F3 which are absolutely correlated, it will divide the weight W among the features as W1,W2,W3 respectively.

We evaluated the Logistic Regression model with L2 regularization to reduce overfitting. The input features are the term frequency inverse document frequency (TF-IDF) values of up to 3 g. This approach results in the model being trained only on this dataset without taking any pre-trained embeddings.

#### Support vector machine (SVM):

Support Vector Machine are a powerful supervised machine learning algorithm used mainly for classification tasks and for regression as well. The goal of an SVM is to find the hyperplane in an N-dimensional space which distinctly classifies the data points (Ekbal and Bandyopadhyay 2008). It means, this algorithm clearly draws the decision boundary line between the data points that belong to a particular category and the ones that do not fall into the category. This is applicable to any kind of data that is encoded as a vector. Therefore, if we could produce appropriate vector representations of the data in our hand, we can use SVM to obtain the desired results (Ekbal and Bandyopadhyay 2008). Here the input features are the same as in LR that is the Term Frequency Inverse Document Frequency (TF-IDF) values of up to 3 g. We evaluate the SVM model with L2 regularization.

#### Multinomial naive bayes (MNB)

This is a Bayesian classifier that works on the naive assumption of conditional independence of features. This means that each input is independent of the other and this is absolutely unrealistic for real data. Nevertheless, it simplifies several complex tasks and hence validates the need.

We evaluate a Naive Bayes classifier for multinomially distributed data, which is derived from Bayes Theorem that finds the probability of a future event given an observed event. MNB is a specialized version of Naive Bayes that is designed more for text documents. Whereas simple naive Bayes would model a document as the presence and absence of particular words, MNB explicitly models the word counts and adjusts the underlying calculations to deal with in. Therefore, the input text data is considered as the bag of words with the count of occurrence of words(frequency) alone considered and the position of words are ignored.

Laplace smoothing is performed using $\alpha =1$ to solve the problem of zero probability and then evaluate the MNB model with TF-IDF vectors.

#### K-nearest neighbour (KNN)

KNN is used for the classification and regression problems but mostly used for classification task.The KNN algorithm stores all available data and classifies, on the basis of similarities, a new data point. This implies that it can be conveniently grouped into a well-suite group using the KNN algorithm as new data emerges. The KNN algorithm assumes that the new upcoming data is related to the available cases and places the new case into the column that is more similar to the categories available. KNN is a non-parametric algorithm as it does not make any assumption on underlying data ((Nongmeikapam et al. 2017)). It is often referred to as a lazy learner algorithm because it does not automatically learn from the training set, but instead stores the dataset and performs an operation on the dataset at the time of classification. At the training point, the KNN algorithm only stores the dataset and then classifies the data into a group that is somewhat close to the current data as it encounters new data.

We use KNN for classification with 3, 4, 5, and 9 neighbours by applying uniform weights.

#### Decision tree (DT)

The decision tree develops models of classification or regression in the context of a tree structure. A dataset is broken down into smaller and smaller subsets, while an associated decision tree is gradually built at the same time. A tree with decision nodes and leaf nodes is the final product. Therefore, a decision tree classification works by generating a tree structure, where each node corresponds to a feature name, and the branches correspond to the feature values. The leaves of the tree represent the classification labels. After sequentially choosing alternative decisions, each node is recursively split again, and finally, the classifier defines some rules to predict the result. Decision trees can accommodate high dimensional data and perform classification without needing much computation. In general, a decision tree classifier has reasonable accuracy. While speaking about its cons, they are vulnerable to mistakes in classification problems having many classes and a comparatively limited number of training examples. Moreover, it is computationally costly for preparation which implies the method of growing a decision tree is expensive in terms of computation. Each candidate splitting area must be organized at each node before it can find the best split. Combinations of fields are used in some algorithms and a search must be made for optimum combination weights. Pruning algorithms can also be costly, because it is important to shape and compare multiple candidate sub-trees. Here, maximum depth was 800, and minimum sample splits were 5 for DT. The criteria were Gini and entropy.

#### Random forest (RF)

Random forest is an ensemble classifier that makes its prediction based on the combination of different decision trees trained on datasets of the same size as training set, called bootstraps, created from a random resampling on the training set itself (Breiman 2001). Once a tree is constructed, a set of bootstraps, which do not include any particular record from the original dataset [out-of-bag (OOB) samples], is used as test set. The error rate of the classification of all the test sets is the OOB estimate of the generalization error. RF showed important advantages over other methodologies regarding the ability to handle highly non-linearly correlated data, robustness to noise, tuning simplicity, and opportunity for efficient parallel processing. Moreover, RF presents another important characteristic: an intrinsic feature selection step, applied prior to the classification task, to reduce the variables space by giving an importance value to each feature. RF follows specific rules for tree growing, tree combination, self-testing and post-processing, it is robust to overfitting and it is considered more stable in the presence of outliers and in very high dimensional parameter spaces than other machine learning algorithms (Caruana and Niculescu-Mizil 2006). We evaluate the RF model with the same features as DT.

#### BERT

BBERT is a language representation model that uses both left and right context conditioning with Masked Language Model training objective in a semi-supervised way (Devlin et al. 2019). These deep contextual representations could be extended to a classification head to fine-tune BERT on downstream NLP tasks. We use BERT with the classification head for classification and fine-tune all parameters in an end to end fashion. We used the huggingface library^11^ to do experiments.

#### CharacterBERT

Many language representation models have adopted the transformers architecture as their fundamental building component due to BERT’s success. Interestingly enough, the wordpiece tokenization in BERT works on most of the NLP tasks, but they are also the reason behind making BERT a complex model in the case of a specialized case. To reduce the complexity, CharacterBERT, a new variation of BERT takes away the wordpiece tokenization entirely and instead utilizes a Character-CNN to represent whole words at the character level over a sub-word level (El Boukkouri et al. 2020). The CharacterBERT is based on the BERT “base-uncased” version (L = 12, H = 768, A = 12, and total parameters = 109.5 M) with follows contains 104.6 M parameters.

#### DistilBERT

DistilBERT is a smaller, cheaper variation of BERT with 40% parameters with 95% of performance from BERT. (Sanh et al. 2019) leveraged pre-trained knowledge distillation along with a smaller language model that achieves similar performances on downstream NLP tasks with less inference time. Knowledge distillation is a compression technique that utilizes a student-teacher model where student i.e. small model learns the behaviour of the teacher i.e. large model with the help of distillation loss.

#### ALBERT

ALBERT (Lan et al. 2019) is a transformer model with fewer parameters than that of BERT trained on self-supervised loss. The foundation of the model is based on the two basic parameter techniques. The first one factorizes embedding parameterization where a large vocabulary embedding matrix is split into small matrices. The second one shares parameters with cross-layers resulting in the reduction of parameters overall. We utilized ALBERT as one of our experiments to study if the claimed performance gain over BERT is observed in our case.

#### RoBERTa

RoBERTA (Liu et al. 2019) unlike BERT is not trained on the next sentence prediction training objective. Instead, larger mini-batches and learning rates are incorporated while training the language model with the Masked Language Modelling objective. RoBERTA with its optimum design choices exceeds the evaluation metric on downstream NLP tasks over the standard BERT baseline. We leveraged the abilities of RoBERTA in our experiments.

#### XLM

XLM (Lample and Conneau 2019) is Cross-lingual Language Model trained on three training objectives: causal language modelling, masked language modelling and translation language modelling. The novelty to this language model comes from the usage of cross-lingual representations and a new supervised learning objective that improves these representations.

#### XLMNet

XLNet use autoregressive (AR) language modeling to estimate the probability distribution of a text corpus while avoiding the usage of the [MASK] token and making concurrent independent predictions. It is accomplished via AR modeling, which gives a logical approach to describe the product rule for factoring the joint probability of the projected tokens.

#### XLM-R

On a number of cross-lingual benchmarks, XLM-RoBERTa was suggested as an unsupervised cross-lingual representation technique that considerably outperformed multi-lingual BERT (Conneau et al. 2020). XLM-R was trained on Wikipedia data from 100 languages and fine-tuned for assessment and inference on a variety of downstream tasks.

**Table 10:** Precision, recall, and F-score for Tamil sentiment analysis

Classifier	Positive	Negative	Mixed feelings	Neutral state	Macro avg	Weighted avg
Support	2503	547	510	631	4402	4402
*Precision*						
SVM	0.57	0.00	0.00	0.00	0.11	0.32
MNB	0.59	0.79	0.46	0.50	0.64	0.59
KNN	0.58	0.19	0.13	0.18	0.34	0.43
DT	0.65	0.32	0.23	0.36	0.42	0.51
LR	0.76	0.36	0.24	0.39	0.36	0.58
RF	0.62	0.59	0.71	0.56	0.66	**0**.**63**
BERT	0.71	0.41	0.41	0.44	0.52	0.59
CharBERT	0.68	0.51	0.31	0.54	0.51	0.59
DistilBERT	0.74	0.41	0.32	0.47	0.51	0.60
ALBERT	0.66	0.39	0.43	0.46	0.52	0.57
RoBERTa	0.70	0.39	0.34	0.46	0.50	0.58
XLM	0.68	0.46	0.44	0.53	0.56	0.60
XLNet	0.68	0.44	0.41	0.42	0.52	0.58
XLM-R	0.71	0.39	0.39	0.52	0.51	0.60
*Recall*						
SVM	1.00	0.00	0.00	0.00	0.20	0.57
MNB	1.00	0.06	0.00	0.04	0.28	0.59
KNN	0.70	0.04	0.06	0.29	0.23	0.46
DT	0.80	0.23	0.14	0.27	0.36	0.55
LR	0.64	0.43	0.28	0.44	0.40	0.54
RF	0.97	0.17	0.02	0.19	0.35	0.62
BERT	0.85	0.39	0.13	0.37	0.46	0.63
CharBERT	0.89	0.27	0.14	0.30	0.45	0.63
DistilBERT	0.80	0.40	0.22	0.44	0.49	0.62
ALBERT	0.88	0.26	0.13	0.32	0.40	0.61
RoBERTa	0.82	0.42	0.12	0.36	0.45	0.61
XLM	0.88	0.32	0.17	0.36	0.45	**0**.**64**
XLNet	0.86	0.32	0.09	0.38	0.42	0.61
XLM-R	0.83	0.44	0.17	0.36	0.47	0.62
*F-score*						
SVM	0.72	0.00	0.00	0.00	0.14	0.41
MNB	0.74	0.11	0.01	0.08	0.28	0.47
KNN	0.63	0.07	0.08	0.23	0.23	0.42
DT	0.72	0.27	0.17	0.31	0.38	0.53
LR	0.69	0.39	0.26	0.41	0.38	0.56
RF	0.76	0.26	0.05	0.28	0.38	0.53
BERT	0.78	0.40	0.20	0.41	0.47	0.60
CharBERT	0.77	0.36	0.20	0.38	0.46	0.59
DistilBERT	0.77	0.40	0.26	0.45	0.50	**0**.**61**
ALBERT	0.75	0.31	0.20	0.38	0.43	0.57
RoBERTa	0.75	0.40	0.18	0.40	0.46	0.58
XLM	0.77	0.38	0.25	0.43	0.48	0.60
XLNet	0.76	0.37	0.15	0.40	0.45	0.58
XLM-R	0.76	0.41	0.24	0.43	0.48	0.60

**Table 11:** Precision, recall, and F-score for Malayalam sentiment analysis

Classifier	Positive	Negative	Mixed feelings	Neutral state	Macro avg	Weighted avg
Support	755	285	131	645	1962	1962
*Precision*						
SVM	0.38	0.00	0.00	0.00	0.08	0.15
MNB	0.49	0.88	0.00	0.60	0.57	0.58
KNN	0.43	0.32	0.41	0.37	0.42	0.41
DT	0.51	0.54	0.35	0.61	0.50	0.54
LR	0.73	0.57	0.34	0.52	0.53	0.59
RF	0.62	0.74	0.56	0.51	0.64	0.61
BERT	0.70	0.34	0.00	0.66	0.49	0.61
CharBERT	0.67	0.62	0.44	0.67	0.64	0.66
DistilBERT	0.65	0.00	0.00	0.60	0.40	0.53
ALBERT	0.44	0.00	0.00	0.33	0.28	0.34
RoBERTa	0.61	0.51	0.17	0.71	0.55	0.61
XLM	0.71	0.53	0.52	0.76	0.65	**0**.**70**
XLNet	0.69	0.52	0.57	0.59	0.63	0.64
XLM-R	0.75	0.49	0.56	0.75	0.65	**0**.**70**
*Recall*						
SVM	1.00	0.00	0.00	0.00	0.20	0.38
MNB	0.92	0.13	0.00	0.45	0.32	0.53
KNN	0.67	0.12	0.12	0.34	0.29	0.41
DT	0.79	0.32	0.21	0.40	0.43	0.53
LR	0.51	0.45	0.32	0.72	0.53	0.57
RF	0.63	0.31	0.14	0.77	0.45	0.58
BERT	0.80	0.27	0.00	0.71	0.50	**0**.**66**
CharBERT	0.81	0.24	0.15	0.71	0.50	0.62
DistilBERT	0.82	0.00	0.00	0.71	0.45	0.64
ALBERT	0.96	0.00	0.00	0.00	0.31	0.45
RoBERTa	0.82	0.30	0.13	0.53	0.49	0.62
XLM	0.82	0.43	0.30	0.50	0.50	0.60
XLNet	0.69	0.36	0.14	0.55	0.49	0.64
XLM-R	0.77	0.63	0.23	0.59	0.50	0.60
*F-score*						
SVM	0.56	0.00	0.00	0.00	0.11	0.21
MNB	0.64	0.23	0.00	0.52	0.31	0.46
KNN	0.53	0.17	0.19	0.36	0.31	0.38
DT	0.62	0.40	0.26	0.49	0.44	0.51
LR	0.60	0.50	0.33	0.60	0.52	0.57
RF	0.62	0.44	0.22	0.62	0.49	0.56
BERT	0.75	0.30	0.00	0.69	0.49	**0**.**63**
CharBERT	0.63	0.35	0.12	0.65	0.49	0.62
DistilBERT	0.73	0.00	0.00	0.65	0.42	0.58
ALBERT	0.60	0.00	0.00	0.01	0.24	0.31
RoBERTa	0.70	0.38	0.15	0.60	0.51	0.60
XLM	0.66	0.48	0.38	0.63	0.62	0.62
XLNet	0.66	0.42	0.14	0.66	0.48	**0**.**63**
XLM-R	0.66	0.55	0.21	0.62	0.48	0.60

**Table 12:** Precision, recall, and F-score for Kannada sentiment analysis

Classifier	Positive	Negative	Mixed feelings	Neutral state	Macro avg	Weighted avg
Support	363	162	57	83	768	768
*Precision*						
RF	0.59	0.70	0.45	0.48	0.55	0.58
SVM	0.47	0.00	0.00	0.00	0.09	0.22
MNB	0.54	0.82	1.00	0.75	0.77	**0**.**68**
KNN	0.51	0.67	0.44	0.50	0.53	0.54
DT	0.59	0.61	0.21	0.39	0.45	0.53
LR	0.70	0.60	0.24	0.38	0.47	0.58
BERT	0.70	0.57	0.32	0.37	0.50	0.59
CharBERT	0.63	0.68	0.24	0.60	0.45	0.54
DistilBERT	0.69	0.50	0.44	0.55	0.47	0.56
ALBERT	0.63	0.55	0.00	0.35	0.42	0.53
RoBERTa	0.66	0.12	0.16	0.26	0.24	0.36
XLM	0.68	0.55	0.26	0.56	0.46	0.51
XLNet	0.71	0.57	0.29	0.49	0.44	0.58
XLM-R	0.56	0.52	0.00	0.46	0.32	0.41
*Recall*						
RF	0.87	0.48	0.06	0.18	0.42	0.59
SVM	1.00	0.00	0.00	0.00	0.20	0.47
MNB	0.99	0.36	0.02	0.04	0.31	0.57
KNN	0.91	0.10	0.07	0.05	0.31	0.52
DT	0.73	0.48	0.19	0.14	0.40	0.54
LR	0.69	0.51	0.26	0.36	0.48	0.57
BERT	0.74	0.58	0.10	0.49	0.50	0.60
CharBERT	0.86	0.53	0.05	0.34	0.47	**0**.**62**
DistilBERT	0.75	0.44	0.22	0.58	0.44	0.57
ALBERT	0.79	0.52	0.00	0.26	0.45	0.59
RoBERTa	0.66	0.46	0.07	0.14	0.27	0.36
XLM	0.66	0.67	0.10	0.37	0.50	**0**.**62**
XLNet	0.64	0.77	0.06	0.40	0.52	0.61
XLM-R	0.75	0.60	0.00	0.07	0.31	0.47
*F-score*						
RF	0.7	0.57	0.11	0.27	0.43	0.55
SVM	0.64	0.00	0.00	0.00	0.13	0.30
MNB	0.70	0.50	0.03	0.07	0.31	0.48
KNN	0.65	0.17	0.12	0.09	0.30	0.43
DT	0.66	0.54	0.20	0.21	0.41	0.52
LR	0.70	0.55	0.25	0.37	0.47	0.57
BERT	0.72	0.57	0.15	0.42	0.49	**0**.**59**
CharBERT	0.72	0.60	0.08	0.43	0.48	**0**.**59**
DistilBERT	0.72	0.47	0.30	0.55	0.45	0.56
ALBERT	0.70	0.58	0.00	0.30	0.43	0.55
RoBERTa	0.66	0.19	0.10	0.18	0.23	0.34
XLM	0.71	0.66	0.18	0.44	0.49	**0**.**59**
XLNet	0.68	0.66	0.10	0.44	0.49	0.58
XLM-R	0.64	0.55	0.00	0.12	0.28	0.41

**Table 13:** Precision, recall, and F-score for Tamil offensive language identification

Classifier	Not-O	O-untargeted	OTI	OTG	OT-Other	Macro avg	Weighted avg
Support	3190	368	315	288	71	4392	4392
*Precision*							
RF	0.77	0.48	0.65	0.43	1.00	0.70	0.72
SVM	0.73	0.67	0.25	0.12	0.00	0.45	0.65
MNB	0.74	0.79	1.00	1.00	0.00	0.75	**0**.**78**
KNN	0.73	0.67	0.25	0.12	0.00	0.45	0.65
DT	0.80	0.29	0.28	0.20	0.11	0.40	0.67
LR	0.87	0.29	0.27	0.14	0.03	0.38	0.71
BERT	0.79	0.29	0.00	0.00	0.00	0.32	0.63
CharBERT	0.83	0.36	0.34	0.32	0.00	0.45	0.71
DistilBERT	0.86	0.38	0.37	0.31	0.00	0.46	0.73
ALBERT	0.79	0.28	0.00	0.00	0.00	0.30	0.63
RoBERTa	0.82	0.43	0.38	0.29	0.00	0.46	0.71
XLM	0.81	0.42	0.38	0.42	0.00	0.47	0.71
XLNet	0.83	0.37	0.37	0.32	0.00	0.42	0.71
XLM-R	0.85	0.40	0.40	0.45	0.00	0.45	0.72
*Recall*							
RF	0.99	0.16	0.06	0.03	0.01	0.31	**0**.**76**
SVM	0.99	0.02	0.01	0.02	0.00	0.19	0.73
MNB	1.00	0.03	0.01	0.00	0.00	0.25	0.74
KNN	0.99	0.02	0.01	0.02	0.00	0.19	0.73
DT	0.92	0.20	0.15	0.12	0.03	0.33	0.72
LR	0.66	0.28	0.30	0.48	0.04	0.41	0.58
BERT	0.96	0.34	0.00	0.00	0.00	0.32	0.75
CharBERT	0.91	0.41	0.28	0.10	0.00	0.40	0.75
DistilBERT	0.88	0.40	0.28	0.35	0.00	0.44	0.75
ALBERT	0.94	0.38	0.00	0.00	0.00	0.31	0.74
RoBERTa	0.93	0.33	0.26	0.13	0.00	0.40	**0**.**76**
XLM	0.94	0.43	0.08	0.15	0.00	0.38	**0**.**76**
XLNet	0.91	0.42	0.27	0.09	0.00	0.41	0.75
XLM-R	0.91	0.47	0.32	0.21	0.00	0.43	0.74
*F-score*							
RF	0.86	0.24	0.12	0.06	0.03	0.33	0.69
SVM	0.84	0.03	0.01	0.03	0.00	0.19	0.63
MNB	0.85	0.06	0.02	0.01	0.00	0.26	0.65
KNN	0.84	0.03	0.01	0.03	0.00	0.19	0.63
DT	0.85	0.24	0.20	0.15	0.04	0.35	0.69
LR	0.75	0.29	0.28	0.22	0.04	0.38	0.63
BERT	0.87	0.31	0.00	0.00	0.00	0.31	0.68
CharBERT	0.87	0.38	0.31	0.15	0.00	0.41	0.73
DistilBERT	0.87	0.39	0.32	0.33	0.00	0.44	**0**.**74**
ALBERT	0.86	0.32	0.00	0.00	0.00	0.30	0.68
RoBERTa	0.87	0.38	0.31	0.18	0.00	0.42	0.73
XLM	0.87	0.43	0.13	0.22	0.00	0.40	0.72
XLNet	0.87	0.39	0.31	0.14	0.00	0.40	0.72
XLM-R	0.88	0.44	0.36	0.28	0.00	0.43	0.73

*O* offensive, *T* targeted, *G* group

**Table 14:** Precision, recall, and F-score for Malayalam offensive language identification

Classifier	Not-O	O-untargeted	OTI	OTG	OT-Other	Macro avg	Weighted avg
Support	1765	29	27	23	–	2001	2001
*Precision*							
RF	0.95	1.00	1.00	1.00	–	0.98	**0**.**95**
SVM	0.88	0.00	0.00	0.00	–	0.18	0.78
MNB	0.89	0.00	0.00	0.00	–	0.36	0.86
KNN	0.95	1.00	1.00	1.00	–	0.97	**0**.**95**
DT	0.95	0.67	0.79	0.65	–	0.78	0.93
LR	0.97	0.50	0.33	0.30	–	0.52	0.91
BERT	0.93	0.00	0.00	0.00	–	0.33	0.88
CharBERT	0.93	1.00	0.00	0.00	–	0.53	0.90
DistilBERT	0.94	0.52	0.22	0.00	–	0.48	0.90
ALBERT	0.93	0.00	0.00	0.00	–	0.33	0.88
RoBERTa	0.94	0.40	0.00	0.00	–	0.43	0.90
XLM	0.92	0.00	0.00	0.00	–	0.33	0.87
XLNet	0.95	0.00	0.02	0.02	–	0.21	0.84
XLM-R	0.71	0.00	0.00	0.00	–	0.35	0.88
*Recall*							
RF	1.00	0.45	0.37	0.39	–	0.58	**0**.**95**
SVM	1.00	0.00	0.00	0.00	–	0.20	0.88
MNB	1.00	0.00	0.00	0.00	–	0.22	0.89
KNN	0.99	0.48	0.44	0.43	–	0.61	**0.95**
DT	0.98	0.55	0.41	0.48	–	0.62	0.94
LR	0.89	0.72	0.56	0.52	–	0.71	0.88
BERT	0.98	0.00	0.00	0.00	–	0.35	0.92
CharBERT	0.97	0.07	0.00	0.00	–	0.35	0.91
DistilBERT	0.96	0.32	0.12	0.00	–	0.43	0.91
ALBERT	0.98	0.00	0.00	0.00	–	0.33	0.92
RoBERTa	0.97	0.28	0.00	0.00	-	0.41	0.92
XLM	0.98	0.00	0.00	0.00	–	0.32	0.91
XLNet	0.64	0.00	0.12	0.10	–	0.20	0.58
XLM-R	0.93	0.00	0.00	0.00	–	0.35	0.88
*F-score*							
RF	0.97	0.62	0.54	0.56	–	0.70	**0**.**94**
SVM	0.94	0.00	0.00	0.00	–	0.19	0.83
MNB	0.94	0.00	0.00	0.00	–	0.23	0.85
KNN	0.97	0.65	0.62	0.61	–	0.72	**0**.**94**
DT	0.97	0.60	0.54	0.55	–	0.68	**0**.**94**
LR	0.93	0.59	0.42	0.38	–	0.59	0.89
BERT	0.95	0.00	0.00	0.00	–	0.34	0.90
CharBERT	0.95	0.13	0.00	0.00	–	0.36	0.90
DistilBERT	0.95	0.40	0.16	0.00	–	0.44	0.90
ALBERT	0.95	0.00	0.00	0.00	–	0.33	0.90
RoBERTa	0.96	0.33	0.00	0.00	–	0.41	0.90
XLM	0.95	0.00	0.00	0.00	–	0.33	0.89
XLNet	0.77	0.00	0.04	0.04	–	0.18	0.69
XLM-R	0.96	0.00	0.00	0.00	–	0.35	0.89

*O* offensive, *T* targeted, *G* group

**Table 15:** Precision, recall, and F-score for Kannada offensive language identification

Classifier	Not-O	O-untargeted	OTI	OTG	OT-Other	Macro avg	Weighted avg
Support	427	33	75	44	14	778	778
*Precision*							
RF	0.65	0.00	0.71	0.43	1.00	0.58	0.63
SVM	0.55	0.00	0.00	0.00	0.00	0.09	0.30
MNB	0.60	0.00	0.86	0.00	0.00	0.37	0.60
KNN	0.61	0.00	0.78	0.67	0.00	0.45	0.60
DT	0.64	0.21	0.57	0.29	0.25	0.42	0.57
LR	0.77	0.04	0.63	0.25	0.22	0.43	0.66
BERT	0.71	0.00	0.45	0.00	0.00	0.32	0.65
CharBERT	0.74	0.00	0.72	0.42	0.00	0.43	0.66
DistilBERT	0.77	0.12	0.46	0.39	0.00	0.40	**0**.**69**
ALBERT	0.71	0.00	0.65	0.00	0.00	0.34	0.62
RoBERTa	0.67	0.00	0.76	0.57	0.00	0.46	0.65
XLM	0.75	0.00	0.79	0.37	0.00	0.43	0.67
XLNet	0.71	0.00	0.64	0.00	0.00	0.34	0.62
XLM-R	0.73	0.00	0.08	0.00	0.00	0.13	0.42
*Recall*							
RF	0.89	0.00	0.35	0.08	0.06	0.32	**0**.**66**
SVM	1.00	0.00	0.00	0.00	0.00	0.17	0.55
MNB	0.98	0.00	0.33	0.00	0.00	0.26	0.62
KNN	0.93	0.00	0.19	0.09	0.00	0.26	0.61
DT	0.78	0.09	0.51	0.18	0.07	0.35	0.60
LR	0.76	0.03	0.59	0.23	0.29	0.43	0.66
BERT	0.84	0.00	0.53	0.00	0.00	0.37	**0**.**71**
CharBERT	0.86	0.00	0.65	0.30	0.00	0.40	**0**.**71**
DistilBERT	0.81	0.04	0.62	0.25	0.00	0.41	0.70
ALBERT	0.87	0.00	0.41	0.00	0.00	0.35	0.70
RoBERTa	0.91	0.00	0.48	0.08	0.00	0.34	0.69
XLM	0.85	0.00	0.59	0.22	0.00	0.40	**0**.**71**
XLNet	0.70	0.00	0.61	0.00	0.00	0.36	0.70
XLM-R	0.74	0.00	0.02	0.00	0.00	0.13	0.41
*F-score*							
RF	0.75	0.00	0.47	0.14	0.11	0.34	0.61
SVM	0.71	0.00	0.00	0.00	0.00	0.12	0.39
MNB	0.74	0.00	0.48	0.00	0.00	0.26	0.54
KNN	0.73	0.00	0.30	0.16	0.00	0.27	0.55
DT	0.70	0.13	0.54	0.22	0.11	0.37	0.58
LR	0.77	0.04	0.61	0.24	0.25	0.43	0.66
BERT	0.81	0.00	0.52	0.00	0.00	0.35	0.68
CharBERT	0.80	0.00	0.64	0.34	0.00	0.41	0.68
DistilBERT	0.80	0.06	0.53	0.29	0.00	0.42	**0**.**69**
ALBERT	0.78	0.00	0.50	0.00	0.00	0.34	0.65
RoBERTa	0.78	0.00	0.59	0.15	0.00	0.36	0.65
XLM	0.79	0.00	0.68	0.28	0.00	0.41	0.68
XLNet	0.78	0.00	0.62	0.00	0.00	0.35	0.66
XLM-R	0.74	0.00	0.03	0.00	0.00	0.13	0.41

*O* offensive, *T* targeted, *G* group

## Results and discussion

The results of the experiments with the classifiers described above for both sentiment analysis and offensive language detection are shown in terms of precision, recall, F1-score and support in Tables 10, 11, 12, 13, 14, and 15.

We used sklearn^12^ to develop the models. A macro-average will compute the metrics (precision, recall, F1-score) independently for each class and average them. Thus this metric treats all classes equally, and it does not take the attribute of class imbalance into account. A weighted average takes the metrics from each class just like a macro average, but the contribution of each class to the average is weighted by the number of examples available for it. The number of comments belonging to different classes from both tasks is listed as the support values in respective tables.

For sentiment analysis, the performance of the various classification algorithms ranges from being inadequate to average on the code-mixed dataset. Logistic regression, random forest classifiers and decision trees were the ones that fared comparatively better across all sentiment classes. To our surprise, we see that SVM performs poorly, having a worse heterogeneity than the other methods. The precision, recall and F1-score are higher for the “Positive” class followed by the “Negative” class. All the other classes performed very poorly. One of the reasons is the nature of the dataset as the classes “Mixed feelings” and “Neutral state” are challenging to label for the annotators owing to the problematic examples described before. It could be observed from Table 12, the highest weighted average precision for sentiment analysis is 0.68 from Multinomial Naive Bayes (MNB), followed by CharBERT and XLM with the highest recall of 0.62, and finally, the highest weighted F-score of 0.59 from multiple classifiers (BERT, CharBERT, XLM).

For offensive language detection, all the classification algorithms perform equally poorly. We see that logistic regression and random forest are the ones that performed relatively better than the others. The precision, recall and F1-score are higher for the “Not Offensive” class followed by the “Offensive Targeted Individual” and “OL” classes. The reasons for the poor performance of other classes are as same as sentiment analysis. From the tables, we see that the classification algorithms have performed better on the task of sentiment analysis in comparison to that of offensive language detection. One of the main reasons could be the differences in the distributions of the classes among the two different tasks. In the case of an Offensive task, we could observe the highest weighted average precision (0.78), recall (0.76) and F-score (0.74) from MNB, RF/RoBERTA/XLM and DistilBERT respectively.

When it comes to the sentiment analysis dataset in Kannada, out of the total of 7671 sentences 46% and 19% belong to the “Positive” and the “Negative” classes respectively while the other classes share 9%, 11% and 15% respectively. This distribution is better when compared to the Kannada dataset for offensive language detection task where 56% belong to “Not Offensive”, while the other classes share a low distribution of 4%, 8%, 6%, 2%, 24%. Although the distribution of offensive and non-offensive classes is skewed in all the languages, we were able to observe that an overwhelmingly higher percentage of comments belonged to non-offensive classes in Tamil and Malayalam datasets than Kannada. 72.4% of comments in Tamil and 88.44% comments in Malayalam datasets were non-offensive while in Kannada only 55.79% of the total comments were non-offensive. This explains why the precision, recall and F-score values of identifying the non-offensive class are consistently higher for Tamil and Malayalam data than Kannada.

Since we collected the posts from movie trailers, we got more positive sentiment than others as the people who watch trailers are more likely to be interested in movies and this skews the overall distribution. However, as the code-mixing phenomenon is not incorporated in the earlier models, this resource could be taken as a starting point for further research. There is significant room for improvement in code-mixed research with our dataset. In our experiments, we only utilized the machine learning methods, but more information such as linguistic information or hierarchical meta-embedding can be utilized.

## Conclusion

This work introduced code-mixed dataset of the under-resourced Dravidian languages. This data set comprises more than 60,000 comments annotated for sentiment analysis and offensive language identification. To improve the research in the under-resourced Dravidian languages, we created an annotation scheme and achieved a high inter-annotator agreement in terms of Krippendorff $\alpha$ from voluntary annotators on contributions collected using Google Form. We created baselines with gold standard annotated data and presented our results for each class in precision, recall, and F-Score. We expect this resource will enable the researchers to address new and exciting problems in code-mixed research. In future work, we intend to investigate whether we can apply these corpora to build corpora for other under-resourced Dravidian languages.

## Footnotes

1 https://github.com/bharathichezhiyan/DravidianCodeMix-Dataset.

2 https://zenodo.org/record/4750858#.YJtw0SYo_0M.

3 Also called Damili or Dramili or Tamil-Brahmi.

4 Keeladi-Book-English-18-09-2019.pdf.

5 Different types of code-mixing are shown in Fig. 2.

6 https://censusindia.gov.in/2011Census/C-16_25062018_NEW.pdf.

7 https://github.com/philbot9/youtube-remarkscraper.

8 https://pypi.org/venture/langdetect/.

9 https://www.nltk.org/.

10 Telugu word for Mr.

11 https://huggingface.co/.

12 https://scikit-learn.org/stable/.

## References

[CR1] Agarwal, A., Xie, B., Vovsha, I., Rambow, O., & Passonneau, R. (2011). Sentiment analysis of twitter data. In: Proceedings of the workshop on language in social media (LSM 2011) (pp. 30–38). Portland, Oregon: Association for Computational Linguistics. https://www.aclweb.org/anthology/W11-0705

[CR2] Agrawal, R., Chenthil Kumar, V., Muralidharan, V., & Sharma, D. (2018). No more beating about the bush: A step towards idiom handling for Indian language NLP. In: Proceedings of the eleventh international conference on language resources and evaluation (LREC-2018). Miyazaki, Japan: European Languages Resources Association (ELRA). https://www.aclweb.org/anthology/L18-1048

[CR83] Andronov, M.S. (1970). Dravidian languages (p. 190). Nauka Publishing House, Central Department of Oriental Literature.

[CR3] Bali, K., Sharma, J., Choudhury, M., & Vyas, Y. (2014). “I am borrowing ya mixing ?” an analysis of English-Hindi code mixing in Facebook. In: Proceedings of the first workshop on computational approaches to code switching (pp. 116–126). Doha, Qatar: Association for Computational Linguistics. 10.3115/v1/W14-3914, https://www.aclweb.org/anthology/W14-3914

[CR4] Barman, U., Das, A., Wagner, J., & Foster, J. (2014). Code mixing: A challenge for language identification in the language of social media. In: Proceedings of the first workshop on computational approaches to code switching (pp. 13–23). Doha, Qatar: Association for Computational Linguistics. 10.3115/v1/W14-3902, https://www.aclweb.org/anthology/W14-3902

[CR5] Blackburn SH (2006). Print, Folklore, and Nationalism in Colonial South India.

[CR6] Breiman L (2001). Random forests. Machine Learning.

[CR7] Caruana, R., & Niculescu-Mizil, A. (2006). An empirical comparison of supervised learning algorithms. In: Proceedings of the 23rd international conference on Machine learning (pp. 161–168)

[CR8] Chakravarthi, B. R. (2020). HopeEDI: A multilingual hope speech detection dataset for equality, diversity, and inclusion. In: Proceedings of the third workshop on computational modeling of people’s opinions, personality, and emotion’s in social media pp 41–53. Association for Computational Linguistics, Barcelona, Spain (Online). https://www.aclweb.org/anthology/2020.peoples-1.5

[CR9] Chakravarthi, B. R., & Muralidaran, V. (2021). Findings of the shared task on hope speech detection for equality, diversity, and inclusion. In: Proceedings of the first workshop on language technology for equality, diversity and inclusion (pp. 61–72). Kyiv: Association for Computational Linguistics. https://www.aclweb.org/anthology/2021.ltedi-1.8

[CR10] Chakravarthi, B. R., Anand Kumar, M., McCrae, J. P., Premjith, B., Soman, K., & Mandl, T. (2020a). Overview of the track on HASOC-offensive Language Identification-DravidianCodeMix. In: Working notes of the forum for information retrieval evaluation (FIRE 2020). CEUR Workshop Proceedings, CEUR-WS. org

[CR11] Chakravarthi, B. R., Jose, N., Suryawanshi, S., Sherly, E., & McCrae, J. P. (2020b). A sentiment analysis dataset for code-mixed Malayalam-English. In: Proceedings of the 1st joint workshop of SLTU (spoken language UDTechnologies for Under-resourced languages) and CCURL (Collaboration and Computing for Under-Resourced Languages) (SLTU-CCURL 2020). Marseille, France: European Language Resources Association (ELRA).

[CR12] Chakravarthi, B. R., Muralidaran, V., Priyadharshini, R., & McCrae, J. P. (2020c). Corpus creation for sentiment analysis in code-mixed Tamil-English text. In: Proceedings of the 1st joint workshop of SLTU (spoken language technologies for under-resourced languages) and CCURL (Collaboration and Computing for Under-Resourced Languages) (SLTU-CCURL 2020). Marseille, France: European Language Resources Association (ELRA)

[CR13] Chakravarthi, B. R., Priyadharshini, R., Muralidaran, V., Suryawanshi, S., Jose, N., Sherly, E., & McCrae, J. P. (2020). Overview of the Track on Sentiment Analysis for Dravidian Languages in Code-Mixed Text. In Forum for information retrieval evaluation (pp. 21–24). New York, NY, USA, FIRE: Association for Computing Machinery. 10.1145/3441501.3441515

[CR14] Chakravarthi, B. R., Priyadharshini, R., Jose, N., Kumar, M. A., Mandl, T., Kumaresan, P. K., Ponnusamy, R., R L H, McCrae, J. P., & Sherly, E. (2021). Findings of the shared task on offensive language identification in Tamil, Malayalam, and Kannada. In: Proceedings of the first workshop on speech and language technologies for dravidian languages (pp. 133–145). Kyiv: Association for Computational Linguistics. https://www.aclweb.org/anthology/2021.dravidianlangtech-1.17

[CR15] Chanda, A., Das, D., & Mazumdar, C. (2016). Unraveling the English-Bengali code-mixing phenomenon. In: Proceedings of the second workshop on computational approaches to code switching (pp. 80–89). Austin, TX: Association for Computational Linguistics. 10.18653/v1/W16-5810, https://www.aclweb.org/anthology/W16-5810

[CR16] Cieliebak, M., Deriu, J. M., Egger, D., & Uzdilli, F. (2017). A twitter corpus and benchmark resources for German sentiment analysis. In: Proceedings of the fifth international workshop on natural language processing for social media (pp. 45–51). Valencia, Spain: Association for Computational Linguistics. 10.18653/v1/W17-1106, https://www.aclweb.org/anthology/W17-1106

[CR17] Clarke, I., & Grieve, J. (2017). Dimensions of abusive language on twitter. In: Proceedings of the first workshop on abusive language online (pp. 1–10). Vancouver, BC, Canada: Association for Computational Linguistics. 10.18653/v1/W17-3001, https://www.aclweb.org/anthology/W17-3001

[CR18] Conneau, A., Khandelwal, K., Goyal, N., Chaudhary, V., Wenzek, G., Guzmán, F., Grave, E., Ott, M., Zettlemoyer, L., & Stoyanov, V. (2020). Unsupervised cross-lingual representation learning at scale. In: Proceedings of the 58th annual meeting of the association for computational linguistics (pp. 8440–8451). Association for Computational Linguistics, Online. 10.18653/v1/2020.acl-main.747, https://www.aclweb.org/anthology/2020.acl-main.747

[CR19] Devlin, J., Chang, M. W., Lee, K., & Toutanova, K. (2019). BERT: Pre-training of deep bidirectional transformers for language understanding. In: Proceedings of the 2019 conference of the North American Chapter of the Association for Computational Linguistics: Human Language Technologies, Vol. 1 (Long and Short Papers) (pp. 4171–4186). Minneapolis, Minnesota: Association for Computational Linguistics. 10.18653/v1/N19-1423, https://www.aclweb.org/anthology/N19-1423

[CR20] Ekbal, A., & Bandyopadhyay, S. (2008). Bengali named entity recognition using support vector machine. NER for South and South East Asian Languages (p. 51)

[CR21] El Boukkouri, H., Ferret, O., Lavergne, T., Noji, H., Zweigenbaum, P., & Tsujii, J. (2020). CharacterBERT: Reconciling ELMo and BERT for word-level open-vocabulary representations from characters. In: Proceedings of the 28th international conference on computational linguistics (pp. 6903–6915). Barcelona, Spain (Online): International Committee on Computational Linguistics. https://www.aclweb.org/anthology/2020.coling-main.609

[CR22] Gai, G. S. (1996). Inscriptions of the early Kadambas. Indian

[CR23] Genkin A, Lewis D, Madigan D (2007). Large-scale Bayesian logistic regression for text categorization. Technometrics.

[CR24] de Gispert, A., Iglesias, G., & Byrne, B. (2015). Fast and accurate preordering for SMT using neural networks. In: Proceedings of the 2015 conference of the North American chapter of the association for computational linguistics: human language technologies (pp. 1012–1017). Denver, Colorado: Association for Computational Linguistics. 10.3115/v1/N15-1105, https://www.aclweb.org/anthology/N15-1105

[CR25] Gitari ND, Zuping Z, Damien H, Long J (2015). A lexicon-based approach for hate speech detection. International Journal of Multimedia and Ubiquitous Engineering.

[CR26] Hu, M., & Liu, B. (2004). Mining and summarizing customer reviews. In: KDD ’04, Proceedings of the tenth ACM SIGKDD international conference on knowledge discovery and data mining (pp. 168-177). New York, NY, USA: Association for Computing Machinery. 10.1145/1014052.1014073

[CR27] Jiang, Q., Chen, L., Xu, R., Ao, X., & Yang, M. (2019). A challenge dataset and effective models for aspect-based sentiment analysis. In: Proceedings of the 2019 conference on empirical methods in natural language processing and the 9th international joint conference on natural language processing (EMNLP-IJCNLP) (pp. 6279–6284). Hong Kong, China: Association for Computational Linguistics. 10.18653/v1/D19-1654, https://www.aclweb.org/anthology/D19-1654

[CR28] Jin, S., & Pedersen, T. (2018). Duluth UROP at SemEval-2018 task 2: Multilingual emoji prediction with ensemble learning and oversampling. In: Proceedings of The 12th international workshop on semantic evaluation (pp. 482–485). New Orleans, Louisiana: Association for Computational Linguistics. 10.18653/v1/S18-1077, https://www.aclweb.org/anthology/S18-1077

[CR29] Jose, N., Chakravarthi, B. R., Suryawanshi, S., Sherly, E., & McCrae, JP. (2020). A survey of current datasets for code-switching research. In: 2020 6th International conference on advanced computing & communication systems (ICACCS)

[CR30] Kouloumpis, E., Wilson, T., & Moore, J. (2011). Twitter sentiment analysis: The good the bad and the OMG! In: Fifth international AAAI conference on weblogs and social media. Citeseer

[CR31] Krippendorff K (1970). Estimating the reliability, systematic error and random error of interval data. Educational and Psychological Measurement.

[CR32] Krishna, P. V., Misra, S., Joshi, D., & Obaidat, M. S. (2013). Learning automata based sentiment analysis for recommender system on cloud. In 2013 International conference on computer (pp. 1–5). IEEE: Information and Telecommunication Systems (CITS).

[CR33] Krishnamurti B (2003). The Dravidian Languages.

[CR34] Kumar, B. S., Thenmozhi, D., & Kayalvizhi, S. (2020). Tamil paraphrase detection using encoder-decoder neural networks. In: International conference on computational intelligence in data science (pp. 30–42). Springer

[CR35] Kumar, R., Ojha, AK., Malmasi, S., & Zampieri, M. (2018). Benchmarking aggression identification in social media. In: Proceedings of the first workshop on trolling, aggression and cyberbullying (TRAC-2018) (pp. 1–11). Santa Fe, New Mexico, USA: Association for Computational Linguistics. https://www.aclweb.org/anthology/W18-4401

[CR36] Lample, G., & Conneau, A. (2019). Cross-lingual language model pretraining. arXiv:1901.07291

[CR37] Lan, Z., Chen, M., Goodman, S., Gimpel, K., Sharma, P., & Soricut, R. (2019). Albert: A lite bert for self-supervised learning of language representations. arXiv preprint arXiv:190911942

[CR38] Liu, Y., Ott, M., Goyal, N., Du, J., Joshi, M., Chen, D., Levy, O., Lewis, M., Zettlemoyer, L., & Stoyanov, V. (2019). Roberta: A robustly optimized bert pretraining approach. arXiv:1907.11692

[CR39] Mæhlum, P., Barnes, J., Øvrelid, L., & Velldal, E. (2019). Annotating evaluative sentences for sentiment analysis: A dataset for Norwegian. In: Proceedings of the 22nd Nordic conference on computational linguistics (pp. 121–130). Linköping University Electronic Press, Turku, Finland. https://www.aclweb.org/anthology/W19-6113

[CR40] Mahadevan, I. (2003). Early tamil epigraphy. From the earliest times to the sixth century ad

[CR41] Mandl, T., Modha, S., Kumar, M. A., & Chakravarthi, B. R. (2020). Overview of the HASOC track at FIRE 2020: Hate speech and offensive language identification in Tamil, Malayalam, Hindi, English and German. Forum for information retrieval evaluation (pp. 29–32). New York, NY, USA, FIRE: Association for Computing Machinery. 10.1145/3441501.3441517

[CR42] Musto, C., de Gemmis, M., Semeraro, G., & Lops, P. (2017). A multi-criteria recommender system exploiting aspect-based sentiment analysis of users’ reviews. In: Proceedings of the eleventh ACM conference on recommender systems (pp 321–325)

[CR43] Ng, AY., & Jordan, M. I. (2002). On discriminative vs. generative classifiers: A comparison of logistic regression and naive bayes. In: Advances in neural information processing systems (pp. 841–848)

[CR44] Nongmeikapam, K., Kumar, W., & Singh, M. P. (2017). Exploring an efficient handwritten Manipuri meetei-mayek character recognition using gradient feature extractor and cosine distance based multiclass k-nearest neighbor classifier. In: Proceedings of the 14th international conference on natural language processing (ICON-2017) (pp. 328–337). Kolkata, India: NLP Association of India. https://www.aclweb.org/anthology/W17-7541

[CR45] Pang, B., & Lee, L. (2004). A sentimental education: Sentiment analysis using subjectivity summarization based on minimum cuts. In: Proceedings of the 42nd annual meeting of the association for computational linguistics (ACL-04) (pp. 271–278). Barcelona, Spain. 10.3115/1218955.1218990, https://www.aclweb.org/anthology/P04-1035

[CR46] Park H (2013). An introduction to logistic regression: From basic concepts to interpretation with particular attention to nursing domain. Journal of Korean Academy of Nursing.

[CR47] Patwa, P., Aguilar, G., Kar, S., Pandey, S., PYKL, S., Gambäck, B., Chakraborty, T., Solorio, T., & Das, A. (2020). Semeval-2020 task 9: Overview of sentiment analysis of code-mixed tweets. In: Proceedings of the 14th International workshop on semantic evaluation (SemEval-2020). Barcelona, Spain: Association for Computational Linguistics

[CR48] Pillai MP (1904). A Primer of Tamil Literature.

[CR49] Pratapa, A., Bhat, G., Choudhury, M., Sitaram, S., Dandapat, S., & Bali, K. (2018). Language modeling for code-mixing: The role of linguistic theory based synthetic data. In: Proceedings of the 56th annual meeting of the association for computational linguistics (Vol. 1: long papers, pp. 1543–1553). Association for Computational Linguistics, Melbourne, Australia. 10.18653/v1/P18-1143, https://www.aclweb.org/anthology/P18-1143

[CR50] Rani, P., Suryawanshi, S., Goswami, K., Chakravarthi, B. R., Fransen, T., & McCrae, J. P. (2020). A comparative study of different state-of-the-art hate speech detection methods for Hindi-English code-mixed data. In: Proceedings of the second workshop on trolling, aggression and cyberbullying. Marseille, France: European Language Resources Association (ELRA)

[CR51] Ranjan, P., Raja, B., Priyadharshini, R., & Balabantaray, R. C. (2016). A comparative study on code-mixed data of Indian social media vs formal text. In: 2016 2nd International conference on contemporary computing and informatics (IC3I) (pp. 608–611). 10.1109/IC3I.2016.7918035

[CR52] Rogers, A., Romanov, A., Rumshisky, A., Volkova, S., Gronas, M., & Gribov, A. (2018). RuSentiment: An enriched sentiment analysis dataset for social media in Russian. In: Proceedings of the 27th international conference on computational linguistics (pp. 755–763). Santa Fe, New Mexico, USA: Association for Computational Linguistics. https://www.aclweb.org/anthology/C18-1064

[CR53] Sakuntharaj, R., & Mahesan, S. (2016). A novel hybrid approach to detect and correct spelling in Tamil text. In: 2016 IEEE international conference on information and automation for sustainability (ICIAfS) (pp. 1–6). IEEE

[CR54] Sakuntharaj, R., & Mahesan, S. (2017). Use of a novel hash-table for speeding-up suggestions for misspelt Tamil words. In: 2017 IEEE international conference on industrial and information systems (ICIIS) (pp. 1–5). IEEE

[CR55] Salomon R (1998). Indian Epigraphy: A Guide to the Study of Inscriptions in Sanskrit, Prakrit, and the Other Indo-Aryan Languages.

[CR56] Sanh, V., Debut, L., Chaumond, J., & Wolf, T. (2019). Distilbert, a distilled version of bert: smaller, faster, cheaper and lighter. arXiv preprint arXiv:191001108

[CR57] Sekhar, AC. (1951). [Evolution of Malayalam]. Bulletin of the Deccan College Research Institute 12(1/2):1–216, http://www.jstor.org/stable/42929457

[CR58] Severyn, A., Moschitti, A., Uryupina, O., Plank, B., & Filippova, K. (2014). Opinion mining on YouTube. In: Proceedings of the 52nd annual meeting of the association for computational linguistics (Vol. 1: long papers, pp 1252–1261). Baltimore, Maryland: Association for Computational Linguistics. 10.3115/v1/P14-1118, https://www.aclweb.org/anthology/P14-1118

[CR59] Shah K, Patel H, Sanghvi D, Shah M (2020). A comparative analysis of logistic regression, random forest and KNN models for the text classification. Augmented Human Research.

[CR60] Shalini, K., Ganesh, H. B., Kumar, MA., & Soman, K. P. (2018). Sentiment analysis for code-mixed Indian social media text with distributed representation. In: 2018 International conference on advances in computing, communications and informatics (ICACCI) (pp. 1126–1131)

[CR61] Sivanantham R, Seran M (2019). Keeladi: An Urban Settlement of Sangam Age on the Banks of River Vaigai.

[CR62] Sowmya Lakshmi, BS., & Shambhavi, B. R. (2017). An automatic language identification system for code-mixed english-kannada social media text. In: 2017 2nd International conference on computational systems and information technology for sustainable solution (CSITSS) (pp. 1–5). 10.1109/CSITSS.2017.8447784

[CR63] Sridhar SN (1978). On the functions of code-mixing in Kannada. International Journal of the Sociology of Language.

[CR64] Sridhar SN, Sridhar KK (1980). The syntax and psycholinguistics of bilingual code mixing. Canadian Journal of Psychology/Revue canadienne de psychologie.

[CR65] Swamy B (1975). The date of Tolkappiyam: A retrospect. Annals of Oriental Research (Madras), Silver Jubilee.

[CR66] Takahashi T (1995). Tamil Love Poetry and Poetics.

[CR67] Thamburaj KP, Rengganathan V (2015). A critical study of spm Tamil literature exam paper. Asian Journal of Assessment in Teaching and Learning.

[CR68] Thamburaj, K. P., Arumugum, L., & Samuel, SJ. (2015). An analysis on keyboard writing skills in online learning. In: 2015 International symposium on technology management and emerging technologies (ISTMET) (pp. 373–377). IEEE

[CR69] Thavareesan, S., & Mahesan, S. (2019). Sentiment analysis in Tamil texts: A study on machine learning techniques and feature representation. In: 2019 14th Conference on industrial and information systems (ICIIS) (pp. 320–325). 10.1109/ICIIS47346.2019.9063341

[CR70] Thavareesan, S., & Mahesan, S. (2020a). Sentiment lexicon expansion using Word2vec and fastText for sentiment prediction in Tamil texts. In: 2020 Moratuwa engineering research conference (MERCon) (pp. 272–276,).10.1109/MERCon50084.2020.9185369

[CR71] Thavareesan, S., & Mahesan, S. (2020b). Word embedding-based Part of Speech tagging in Tamil texts. In: 2020 IEEE 15th International conference on industrial and information systems (ICIIS) (pp. 478–482). 10.1109/ICIIS51140.2020.9342640

[CR72] Thenmozhi D, Aravindan C (2018). Ontology-based Tamil-English cross-lingual information retrieval system. Sādhanā.

[CR73] Thottingal, S. (2019). Finite state transducer based morphology analysis for Malayalam language. In: Proceedings of the 2nd workshop on technologies for MT of low resource languages. European Association for Machine Translation (pp. 1–5). Dublin, Ireland. https://www.aclweb.org/anthology/W19-6801

[CR74] Tian, Y., Galery, T., Dulcinati, G., Molimpakis, E., & Sun, C. (2017). Facebook sentiment: Reactions and emojis. In: Proceedings of the fifth international workshop on natural language processing for social media (pp. 11–16). Association for Computational Linguistics, Valencia, Spain, 10.18653/v1/W17-1102, https://www.aclweb.org/anthology/W17-1102

[CR75] Vikram TN, Urs SR (2007). Development of Prototype Morphological Analyzer for he South Indian Language of Kannada.

[CR76] Vyas, Y., Gella, S., Sharma, J., Bali, K., & Choudhury, M. (2014). POS tagging of English-Hindi code-mixed social media content. In: Proceedings of the 2014 conference on empirical methods in natural language processing (EMNLP) (pp. 974–979). Doha, Qatar: Association for Computational Linguistics. 10.3115/v1/D14-1105, https://www.aclweb.org/anthology/D14-1105

[CR77] Wiebe J, Wilson T, Cardie C (2005). Annotating expressions of opinions and emotions in language. Language Resources and Evaluation.

[CR78] Wilson, T., Wiebe, J., & Hoffmann, P. (2005). Recognizing contextual polarity in phrase-level sentiment analysis. In: Proceedings of human language technology conference and conference on empirical methods in natural language processing (pp. 347–354). Vancouver, British Columbia, Canada: Association for Computational Linguistics. https://www.aclweb.org/anthology/H05-1044

[CR79] Winata, G. I., Lin, Z., & Fung, P. (2019). Learning multilingual meta-embeddings for code-switching named entity recognition. In: Proceedings of the 4th workshop on representation learning for NLP (RepL4NLP-2019) (pp 181–186). Florence, Italy: Association for Computational Linguistics. 10.18653/v1/W19-4320, https://www.aclweb.org/anthology/W19-4320

[CR80] Zampieri, M., Malmasi, S., Nakov, P., Rosenthal, S., Farra, N., & Kumar, R. (2019). Predicting the type and target of offensive posts in social media. In: Proceedings of the 2019 conference of the North American chapter of the association for computational linguistics: Human language technologies (Vol. 1, pp. 1415–1420) (long and short papers). Minneapolis, Minnesota: Association for Computational Linguistics. 10.18653/v1/N19-1144, https://www.aclweb.org/anthology/N19-1144

[CR81] Zampieri, M., Nakov, P., Rosenthal, S., Atanasova, P., Karadzhov, G., Mubarak, H., Derczynski, L., Pitenis, Z., & Çöltekin, c. (2020). SemEval-2020 Task 12: Multilingual offensive language identification in social media (OffensEval 2020). In: Proceedings of SemEval

[CR82] Zvelebil KV (1991). Comments on the Tolkappiyam theory of literature. Archív Orientální.

